# Unidirectional cellulose nanocrystal hydrogel for bio-based invertible chiral optics and sensors

**DOI:** 10.1038/s41467-026-73859-7

**Published:** 2026-06-03

**Authors:** Yi-Tao Xu, Zongzhe Li, M. Andrea Ortiz Medrano, Mark J. MacLachlan

**Affiliations:** 1 Department of Chemistry, University of British Columbia, Vancouver, BC Canada; 2 Stewart Blusson Quantum Matter Institute, University of British Columbia, Vancouver, BC Canada; 3 WPI Nano Life Science Institute, Kanazawa University, Kanazawa, Japan; 4 Bioproducts Institute, University of British Columbia, Vancouver, BC Canada

**Keywords:** Gels and hydrogels, Gels and hydrogels, Bioinspired materials, Synthesis and processing

## Abstract

Realizing invertible chiral optical properties in the visible region is crucial for developing optical systems with applications in displays and sensors. Achieving this with cellulose nanocrystal (CNC)-based materials is difficult because their chiral nematic structures are inherently fixed in a left-handed configuration. Here, we demonstrate that by introducing a unidirectional (or monodomain nematic-like) CNC hydrogel as a retardation layer onto a chiral nematic CNC film, the naturally left-handed chiral reflections of CNC films can be converted to right-handed reflections. Furthermore, the right-handed reflection can be switched back to their left-handed state simply by applying pressure. These mechano-responsive invertible chiral optical phenomena, along with the manipulation of light through the cooperation of unidirectional and chiral nematic CNC structures, opens up new opportunities for developing bio-based invertible chiral optics and sensors.

## Abstract (web summary)

Designing systems with invertible optical properties is attractive for developing optical systems for displays and sensors. Here the authors design a cellulose nanocrystal-based hydrogel that controls the handedness of light for switchable optical properties.

## Introduction

Selective diffraction of circularly polarized (chiral) light in the visible region is attractive for applications in encryption, displays, and sensors^1–6^. One strategy for producing visible chiral reflections is by using chiral nematic structures^5,6^. These structures are commonly found in the chiral nematic (or cholesteric) liquid crystalline phase, which comprises pseudolayers of aligned molecules (or polymers, nanoparticles) whose orientation rotates about a single axis, known as the helical axis^5,7^. When the helical pitch matches the wavelength of visible light, chiral nematic structures selectively reflect light with a handedness determined by the helical orientation of the material, producing vivid colors^5,6^. In nature, the exoskeletons of certain beetles like *Cetonia aurata* have a biopolymer matrix in which chitin nanofibrils organize into a chiral nematic structure, giving them their metallic green appearance^6^.

Cellulose nanocrystals (CNCs) are renewable and biodegradable materials obtained from acid hydrolysis of biomass^8^. They are nanorod-like, and can form stable suspensions in water due to the negative surface charges imparted by sulfate half-ester groups^8^. An important property of CNCs is their ability to form a lyotropic liquid crystal with a left-handed (L) chiral nematic structure in water^9,10^. This characteristic, combined with their sustainability, makes CNCs highly attractive for developing eco-friendly materials that produce visible readouts while also responding to environmental changes. CNCs have been combined with hydrogels, elastomers, and other materials to create stimuli-responsive CNC-based chiral optical systems^11–18^. These efforts have resulted in photonic materials where the wavelength of circularly polarized light can be tuned across the entire visible spectrum in response to stimuli such as ionic strength^12^, mechanical force^11,16^, temperature^14^, and more^13,15,18^.

However, controlling the handedness of the reflected light, which could enable switching between L and right-handed (R) chiral reflections, remains a challenge. This difficulty arises because the helical organization of CNC-based materials is inherently fixed in an L configuration. In contrast, some natural materials with L chiral nematic structures can still produce R chiral reflections. For example, the exoskeletons of the golden scarab beetle *Chrysina resplendens* (or *Plusiotis resplendens*) have a chitinous layer with L-chiral nematic organization separated by a unidirectional chitin layer, which is analogous to the monodomain nematic-like liquid crystalline phase and acts as a wave plate to retard (delay) one component of polarization with respect to its orthogonal component^19,20^. On passing through this unidirectional chitin layer, the reflected L-circularly polarized light from the lower L-chiral nematic layers (i.e., chiral nematic organization below the unidirectional layer) is converted into R circularly polarized reflection, while the upper L chiral nematic layers continue to reflect L circularly polarized light^19,20^. Therefore, *Chrysina resplendens* can display both L and R chiral reflections. Recently, Godinho and coworkers mimicked this natural system by introducing a thermotropic nematic liquid crystalline layer within the chiral nematic organization of CNCs, enabling controllable R chiral reflections in response to heat or electric field^20^. Svagan, Jonsson and coworkers used a photoresponsive liquid crystal to modulate the optical response of CNC films to obtain light reflection with both handedness in response to UV light^21^. Other research efforts have explored the introduction of cellulose layers with multi-domain nematic-like structures, layer-by-layer assembly, controlled evaporation of CNC suspensions with rotation, and the use of birefringent tape to achieve R chiral reflection^22–28^. However, these strategies still result in the simultaneous reflection of L and R circularly polarized light, or fail to produce visible colors. In the visible region, achieving R chiral reflections while significantly suppressing L chiral reflections in CNC-based materials across a wide range of visible spectrum remains an unsolved challenge.

To overcome this limitation, a potential approach is to design a unidirectionally ordered, elastic material that functions as a responsive broadband retardation plate. Crucially, these materials should introduce appropriate phase retardation across a wide visible-wavelength range to convert L circularly polarized reflection from the CNC-based chiral nematic materials of varying color into an R chiral reflection, with minimal residual L reflection. Some existing unidirectionally organized materials, such as birefringent tape, unidirectional hydrogels, elastomers, and others exhibit visible interference colors between crossed polarizers^28–31^, indicating their potential to introduce phase shifts for controlling the chiral reflection at specific wavelengths. However, they lack broadband performance, and their inability to introduce an appropriate phase shift across the visible range is a significant limitation. This deficiency prevents the desired handedness inversion of reflected light from CNC-based chiral nematic materials, resulting in simultaneous L and R chiral reflections.

Here, we develop a unidirectional CNC hydrogel as a responsive retardation layer, capable of introducing a phase shift of ~ 0.33 – 0.65 waves over a broad range of visible wavelengths, enabling the conversion of L chiral reflections from chiral nematic CNC films into R chiral reflections. The optical properties of this bio-based unidirectional structure are pressure-sensitive, likely due to thickness variations or perturbation in the structural order. Building on this, we developed a model system comprising a chiral nematic CNC film and a unidirectional CNC hydrogel that achieves invertible chiral reflections over a wide visible-wavelength range. These materials have potential applications for optical encryption or mechano-responsive invertible chiral optical sensors.

## Results

### Preparation and optical properties of the unidirectional CNC hydrogels

**Fig. 1:**
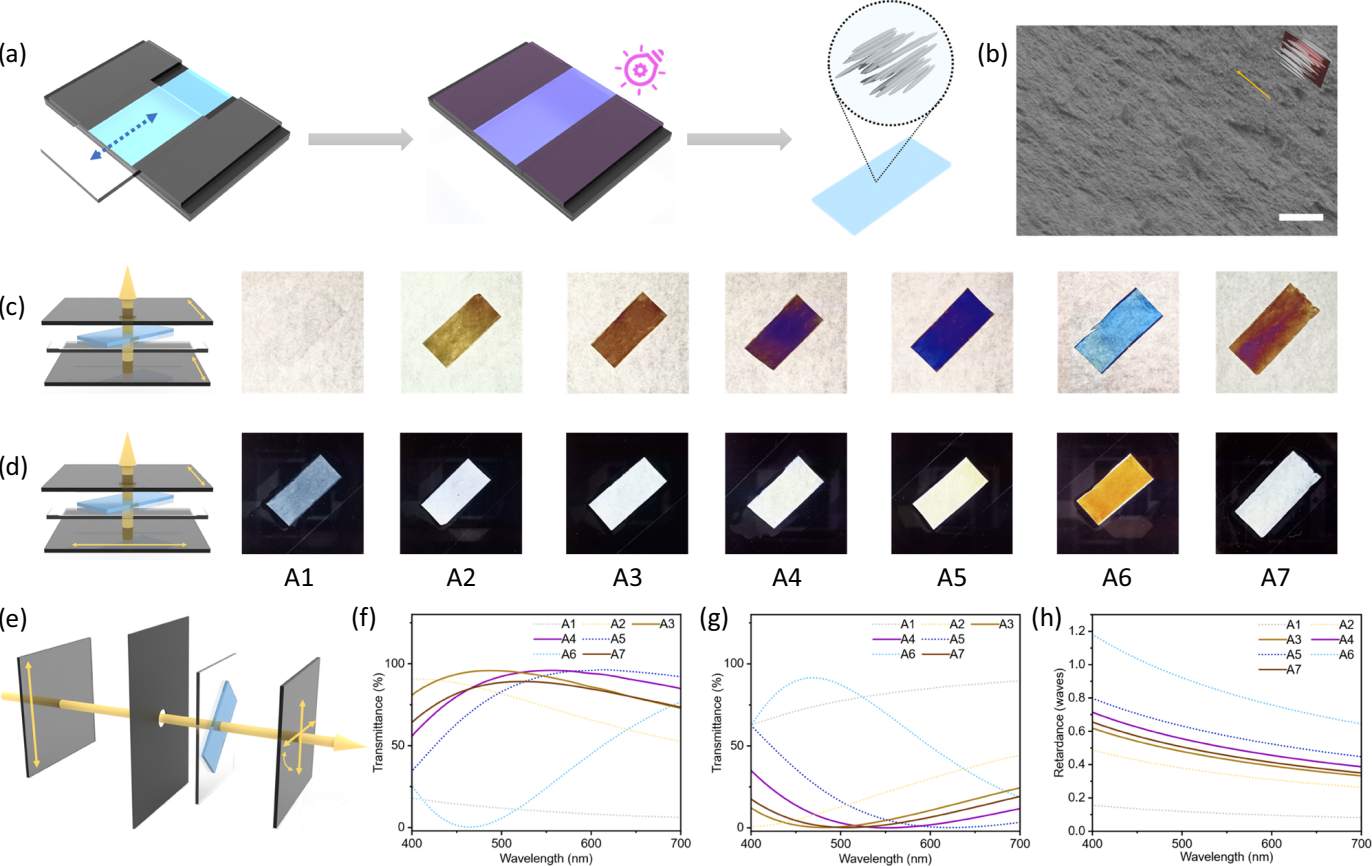
Polarized light observation of the unidirectional CNC hydrogels. **a** Schematic illustration of the preparation of the unidirectional CNC hydrogels via shearing and photopolymerization. **b** Cross-sectional SEM images of the air-dried unidirectional CNC hydrogel (A3). Scale bar is 1 μm. Transmission images of A1–A7 under (**c**) parallel and (**d**) crossed polarizers, with the shear direction oriented at 45° relative to the polarization axis of either polarizer. **e** Schematic of the experimental setup for measuring the transmittance of A1–A7 under crossed and parallel polarizers with their polarizing axis fixed at 45° relative to the shear direction. **f**, **g** Transmission spectra of A1–A7 measured under (**f**) parallel and (**g**) crossed polarizers. **h** Spectral retardance for A1–A7. The length of the hydrogels shown is ~ 2.3 cm.

To obtain a unidirectional hydrogel, a precursor mixture was prepared by combining CNCs, clay nanosheets, monomer, cross-linker, and photoinitiator; clay nanosheets were introduced to increase the viscosity of the mixture. The precursor mixture was drop-cast onto a custom setup as illustrated in Fig. 1a and Supplementary Fig. 1, and sheared by repeatedly moving a capping slide back and forth. Subsequent UV light-triggered photopolymerization afforded a transparent CNC hydrogel. Samples A1–A7 were prepared, where the concentration of the components varied slightly (the composition of each hydrogel can be seen in Supplementary Table 1). Rheology experiments (Supplementary Fig. 2) showed that introducing clay nanosheets increased the complex viscosity and loss/storage modulus of the precursor, suggesting a more flow-resistant, elastically reinforced matrix that can restrict particle rotational/translational mobility and thus help preserve shear-induced orientation during subsequent polymerization. During polymerization, the clay-filled system maintained higher moduli, reflecting progressive network formation in which clay promotes earlier, and stronger development of an elastic network compared with the clay-free formulation. Cross-sectional SEM images (Fig. 1b and Supplementary Fig. 3) revealed a unidirectional texture consistent with the alignment of CNCs within the hydrogel. The 2D-XRD pattern showed an azimuthal angle dependence on the diffraction intensity; the corresponding Hermans orientation factor (S) of ~ 0.38 indicated moderate alignment of CNCs within the hydrogel (Supplementary Fig. 4). Transmission photographs of the CNC hydrogels (Fig. 1c, d), taken in the presence of polarizers with their polarization axes fixed at 45° relative to the shear direction, displayed a monochromatic color, typical of unidirectional CNC alignment.

Interestingly, by carefully adjusting the particle concentration or hydrogel thickness, the unidirectional hydrogels (A2–A5 and A7) can exhibit a uniform white transmission color (Fig. 1d) when viewed between crossed polarizers (the hydrogel was placed at 45° between two crossed polarizers). The corresponding transmission spectra show a broad peak spanning the visible region. These results suggest that these hydrogels can rotate the polarization of incident linearly polarized light over a broad range of wavelengths, enabling light of various wavelengths within the visible spectrum to pass through the second polarizer (i.e., the analyzer, Fig. 1f). Further increasing the total particle concentration gives hydrogels (A6) that show interference color at specific wavelengths, indicating that the polarization rotation of incident linearly polarized light occurs over relatively narrower spectral bands.

To further study this property, the polarizer was rotated to achieve a parallel polarizer configuration. Under this condition, hydrogel A2, A3, A4, and A7 exhibit a brown transmission color, while A5 displays a dark blue color (Fig. 1c). The corresponding transmission spectra show a significant reduction in transmission intensity for a broad range of visible wavelengths (Fig. 1g). This implies that the linearly polarized light is partially blocked due to polarization rotation after passing through the hydrogel (A2, A3, A4, and A7). This behavior is analogous to that of a broadband half-wave plate, which rotates the polarization of incident linearly polarized light by 2 × 45° (the incident polarization is at an angle of 45° to the plate’s axis) across a wide range of wavelengths, allowing the transmitted polarization to be either parallel or perpendicular to the analyzer.

To assess the influence of unidirectional hydrogels on the phase shift between polarization components, we calculated the wave retardation^32,33^. A wave retardation of ~0.33 to 0.65 waves (0.66 π–1.30 π) across the visible spectrum was observed for hydrogels A3, A4, and A7 (Fig. 1h). We assume that these hydrogels, exhibiting such optical characteristics, could function similarly to the monodomain nematic-like layer found in *Chrysina resplendens*, capable of converting left-handed circularly polarized light (L-CPL) into right-handed chiral light throughout the visible region. This conversion is expected since a phase shift (φ) within the range of π/2 < *φ* < 3π/2 typically leads to the transformation of L circularly polarized reflections into R circularly polarized reflections (analysis is provided in Supplementary Information). Other hydrogels (i.e., A1, A2, A5, and A6) cause retardation that is very close to 0.25 or 0.75 waves, or fall outside the 0.25–0.75 wave range in part of the visible region, which may limit their effectiveness for the inversion of chiral light over a broad range of wavelengths.

### Conversion of L circularly polarized reflections of chiral nematic CNC films into R chiral reflections

**Fig. 2:**
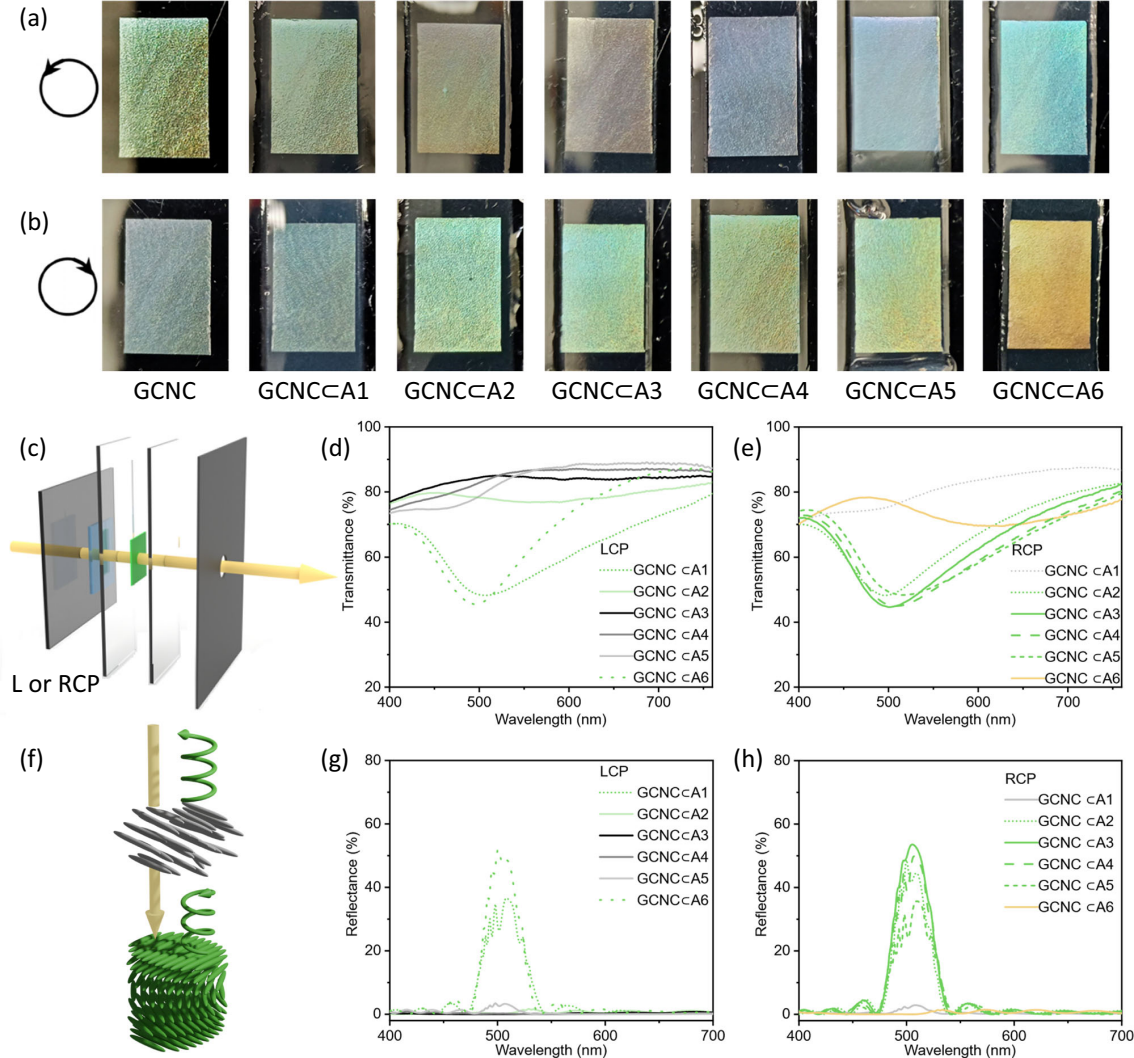
Observation of R chiral reflection in chiral nematic CNC films coupled with unidirectional CNC hydrogels. Photographs of the chiral nematic CNC films with and without the unidirectional CNC hydrogels (A1-A6) on top; photos are taken through a (**a**) LCP and (**b**) RCP. **c** Schematic illustration of the setup for L-CPL or R-CPL transmission measurements of GCNC⊂A1, GCNC⊂A2, GCNC⊂A3, GCNC⊂A4, GCNC⊂A5, and GCNCA⊂6; normal light is circularly polarized by LCP or RCP. L-CPL (**d**) and R-CPL (**e**) transmission spectra of GCNC⊂A samples. **f** Schematic illustration of the handedness inversion of the green reflected light after passing through the unidirectional hydrogel. Simulated L-CPL (**g**) and R-CPL (**h**) reflectance spectra using Berreman’s method. The width of the GCNC film is ~ 1.1 cm.

To examine the unidirectional hydrogel’s ability to invert circularly polarized reflections, hydrogels were placed onto the top of a green CNC film (GCNC) with a glass slide in between for direct observation through a circular polarizer (Fig. 2a, b). Samples are designated as GCNC⊂A, where “*⊂*“ means A is positioned atop the CNC film, separated by a glass slide (BCNC and RCNC for blue and red CNC film, respectively. A can be A1, A2, A3, A4, A5, A6 or A7. Circularly polarized light (CPL) transmission spectra were then measured for these samples via UV–visible spectroscopy, using circularly polarized incident light generated with a left-handed circular polarizer (LCP) or a right-handed circular polarizer (RCP) (Fig. 2c, d and e). As anticipated, the hydrogel that introduces a phase shift of <0.25 waves (A1) in the green spectral region (500–560 nm) does not alter the handedness of reflected light. The green color of GCNC⊂A1 remains visible under an LCP and disappears under an RCP, consistent with the L-CPL reflection observed in the green chiral nematic CNC films (GCNC). Conversely, when hydrogel A2, which introduces a phase shift of 0.33 to 0.38 waves in the green spectral region (500–560 nm), was placed on top of GCNC, GCNC⊂A2 appeared very pale green under an LCP but retained its green color when viewed through an RCP. The corresponding L-CPL transmission spectrum showed only a small reflection peak, whereas a distinct reflection peak was observed when these samples were illuminated with R-CPL. This is a unique example of producing visible R chiral reflections while significantly suppressing L chiral reflections in CNC-based materials. It also supports our previous thinking that unidirectional CNC hydrogels introducing a phase shift between 0.25 and 0.75 waves can cause handedness inversion in the circularly polarized light (Fig. 2f).

This handedness inversion of light is more obvious with A3 and A4 (Fig. 2d, e). Samples GCNC⊂A3 and GCNC⊂A4 appear nearly colorless when viewed under an LCP. The corresponding transmission spectra measured using left-handed circularly polarized light show almost no peaks for GCNC⊂A3 and GCNC⊂A4. Instead, these samples display their green color when viewed through an RCP. By looking into the phase shift introduced by these unidirectional hydrogels, it can be seen that A3 and A4 introduce phase shifts of 0.42 < *φ* < 0.55 waves in the green spectral region—closer to a half wave than that introduced by A2. This suggests that unidirectional hydrogels capable of inducing phase shifts closer to a half-wave across a broad range of wavelengths are more effective at producing a pronounced handedness inversion effect for the reflected light of chiral nematic CNC films, exhibiting a stronger helical twist.

In addition to the visible inversion of circularly polarized light, we observed that combining the green CNC film (GCNC) with hydrogel A6 enabled the light reflection of both left- and right-handed circularly polarized light. Sample GCNC⊂A6 appears cyan when viewed under an LCP and orange under an RCP, in contrast to the green color seen in the bare CNC films (GCNC) or GCNC⊂A1 to GCNC⊂A5 samples. This phenomenon can be ascribed to the retardance differences of A6 across the visible spectrum. In the orange and red spectral region (610–700 nm), the phase shift introduced by A6 is >0.25 waves, which results in hydrogel A6 altering the handedness of light. In the blue–green region (400–560 nm), the phase shift is >0.75 waves, so the reflection in this region remains left-handed and the reflection peak narrows, producing the distinct cyan color. The combined L and R circularly polarized reflection resembles that of the bare CNC film (Supplementary Fig. 5), supporting the above analysis that hydrogel A6 only alters the handedness of certain wavelengths of light (i.e., light in the orange and red spectral region). As a comparison, hydrogel A5 introduces a phase shift of ~ 0.44 to 0.75 waves over 425–700 nm, enabling the inversion of green chiral reflection from the underlying CNC film rather than producing light reflection of both left and right handedness. This phenomenon from GCNC⊂A6 is of interest as it shows that the reflection color of both left and right handedness could be achieved and controlled by introducing a unidirectional structure with selective retardance for specific wavelengths. Furthermore, responsive reflection colors across the visible spectrum may be realized if the phase shift introduced by the hydrogel could be dynamically adjusted, though this requires additional future studies.

We also performed experiments to evaluate the effect of water loss on the chiral optical response. Water loss via evaporation or osmosis can shrink the hydrogel (Supplementary Fig. 6). After ~2.5 h evaporation at room temperature, A3 lost its broadband polarization-rotation feature and became more wavelength-selective, resembling A6. Consequently, handedness inversion occurred only over a limited wavelength range, producing reflection in both left- and right-handed channels, similar to GCNC⊂A6. A similar trend was observed after treatment with 1 mM NaCl solution (osmotic dehydration). Nevertheless, the loss of the broadband feature was reversible upon re-swelling the hydrogel in water.

To also visualize how the retardation layer with varying Δn affects the chiral reflection from the ideal chiral nematic stacks (e.g., without defects or pitch variations), we performed numeric simulation (see Section 3 in Supplementary Information for details) by means of the 4 × 4 Berreman matrix for a multilayer model (air │ uniaxial materials │ chiral nematic stacks │ silica substrate)^34–36^. Note that *n*_0_ and *n*_e_ for defining the uniaxial materials are unknown; however, since Δn can be derived from Cauchy approximation (Supplementary Fig. 7), we manually set *n*_0_ = 1.3 and *n*_e_ = 1.3 + Δn. The simulated spectra (Fig. 2g, h) predict that A2, A3, A4 and A5 effectively invert the chiral reflection from the underlying chiral nematic stacks with markedly increased right-handed reflectance and strongly suppressed left-handed components, whereas A1 and A6 do not invert the chiral reflection, in agreement with the experimental measurements. The simulated reflection bands are noticeably narrower than the experimental spectra. This can be ascribed to the CNC film’s heterogeneities, including defect formation and pitch-length variations across the film thickness (e.g., due to drying dynamics, gravity-driven gradients or flow effects), which broaden the chiral nematic stop band and yield a wider peak in measurements (this broadening also contributes to the observed orange reflection under RCP for GCNC⊂A6).

**Fig. 3:**
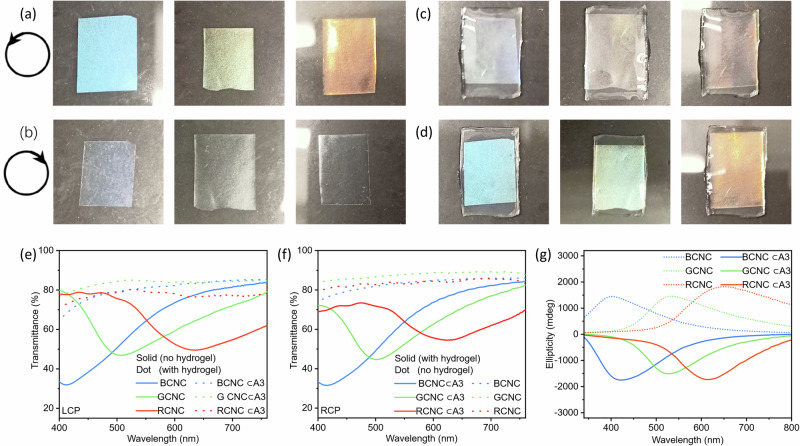
Controlling the handedness of reflected light with RGB colors using CNC-based hydrogel materials. Photographs of the chiral nematic CNC films with blue (BCNC), green (GCNC), and red (RCNC) reflection colors taken through an LCP (**a**) and RCP (**b**). Photographs of BCNC⊂A3, GCNC⊂A3 and RCNC⊂A3 taken through an LCP (**c**) and RCP (**d**) with the unidirectional hydrogel A3 coated on top. L-CPL (**e**) and R-CPL (**f**) transmission spectra of samples shown in (**a**–**d**). **g** CD spectra of BCNC, GCNC, RCNC, BCNC⊂A3, GCNC⊂A3 and RCNC⊂A3. The film widths are approximately 1.0 cm for BCNC, 0.8 cm for GCNC, and 1.1 cm for RCNC.

In the next experiments, we were interested in observing if the unidirectional CNC hydrogels could realize handedness inversion of reflected light for chiral nematic CNC films with different reflection colors. We selected A3 for this study as it can introduce a phase shift of 0.33 < *φ *< 0.61 waves across almost the entire visible spectrum. Blue, green and red CNC films were prepared, and their combination with A3 exhibited visible colors under an RCP, opposite to their behaviors without the hydrogel (Fig. 3). This observable invertible chiral optical property was also examined by CPL transmission (Fig. 3e, f) and CPL reflectance measurements (Supplementary Fig. 8), which show an obvious reflection peak when irradiated with L-CPL; this peak is almost absent when R-CPL is used. The circular dichroism (CD) spectra (Fig. 3g) show intense negative signals with slight peak shifts for BCNC⊂A3, GCNC⊂A3, and RCNC⊂A3—a reversal of the behavior seen in the bare CNC films. This observable handedness inversion of reflected light indicates that unidirectional hydrogels capable of introducing appropriate phase shifts over a broad range of wavelengths are important in controlling the handedness of reflected light from CNC films with RGB colors.

**Fig. 4:**
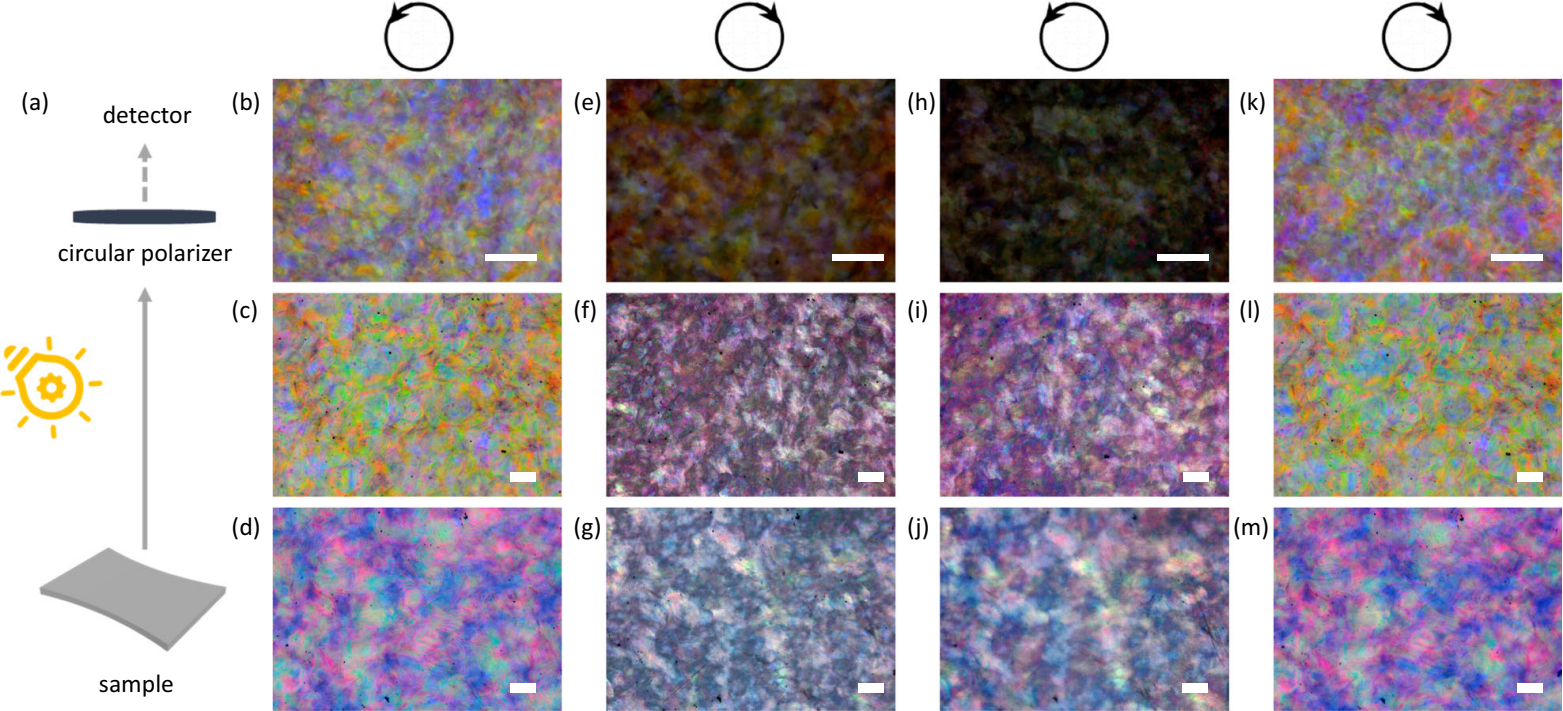
Microscopic observation of CNC chiral nematic films with and without hydrogels under a circular polarizer. **a** Schematic of the setup for reflection circularly polarized optical microscopy (CPOM) characterization. The setup was modified from conventional polarized optical microscopy by replacing the linear polarizers with a circular polarizer. CPOM images of BCNC (**b**), GCNC (**c**) and RCNC (**d**) with LCP. CPOM images of BCNC (**e**), GCNC (**f**) and RCNC (**g**) with RCP. CPOM images of BCNC⊂A3 (**h**), GCNC⊂A3 (**i**) and RCNC⊂A3 (**j**) with LCP. CPOM images of BCNC⊂A3 (**k**), GCNC⊂A3 (**l**) and RCNC⊂A3 (**m**) with RCP. Scale bar: 100 µm.

We were also interested in observing this inverted chiral optical property across the wide visible region enabled by A3 at the microscale. Reflection circularly polarized optical microscopy (CPOM), which is achieved by replacing the linear polarizer with circular polarizer as illustrated in Fig. 4a, was employed for this purpose. CPOM images show the microdomains of CNC films combined with unidirectional CNC hydrogel is much brighter under an RCP compared to an LCP (Fig. 4h–m)—opposite to the phenomenon observed in CNC films without hydrogels (Fig. 4b–g), indicating that the reflected light is inverted after passing through the hydrogel so that it passes through the RCP rather than the LCP.

### Photonic patterns based on the unidirectional CNC hydrogels and chiral nematic CNC films

**Fig. 5:**
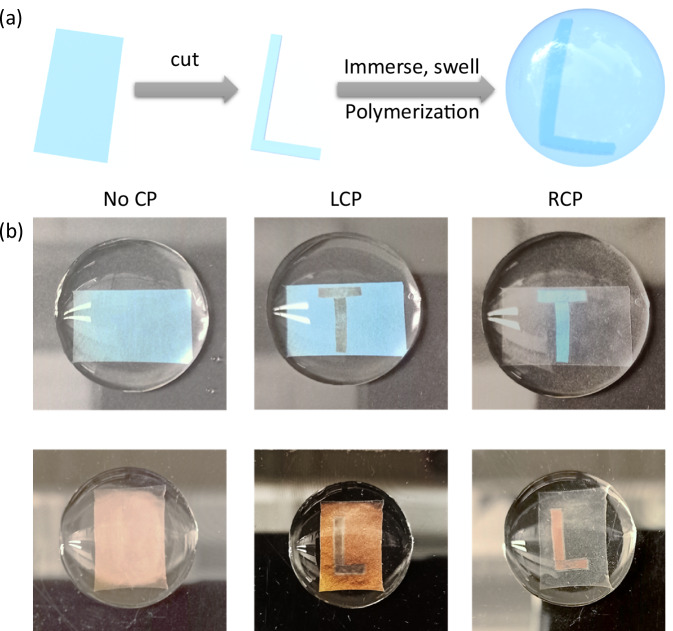
Photonic patterns observed through a circular polarizer (CP). **a** Schematic illustration of the encapsulation of a unidirectional hydrogel pattern within a disordered hydrogel matrix. **b** Photographs showing the patterns without CP (left), under LCP (center) and RCP (right). The width of the films is ~ 1.0 cm.

Given the emergence of visible R chiral reflections from CNC chiral nematic films upon adding a layer of the unidirectional CNC hydrogel, we sought to take advantage of this inverted optical property to create photonic patterns recognizable with a circular polarizer, which may have potential applications in optical encryption. The unidirectional hydrogel patterns from A3, shaped like the letters “L” and “T”, can be fabricated by cutting a bulk hydrogel into the desired shapes or fitting small hydrogel strips together. The pattern was then immersed in a hydrogel precursor solution (water containing a monomer, cross-linker, and photoinitiator; see Supplementary Information for details). After swelling, the solution was polymerized to form a disordered hydrogel matrix encapsulating the unidirectional hydrogel patterns. (Fig. 5a). The resulting hydrogels with “L” and “T” patterns were placed onto blue and red CNC films, respectively, with a glass slide separating the hydrogels from the films. A few drops of water were added to the hydrogel surface to maintain hydration and decrease the visibility of patterns during photography. When viewed through LCP, regions containing the “T” hydrogel pattern exhibited diminished blue color, while the surrounding disordered hydrogel regions appeared blue. This resulted in a distinct “T” pattern recognizable by LCP (Fig. 5b). This phenomenon was entirely reversed when observed under an RCP. Additionally, similar results were achieved with red CNC films, demonstrating that this hydrogel-based chiral photonic patterning approach is applicable to other visible wavelengths.

### CNC-based mechano-responsive invertible chiral optical systems

**Fig. 6:**
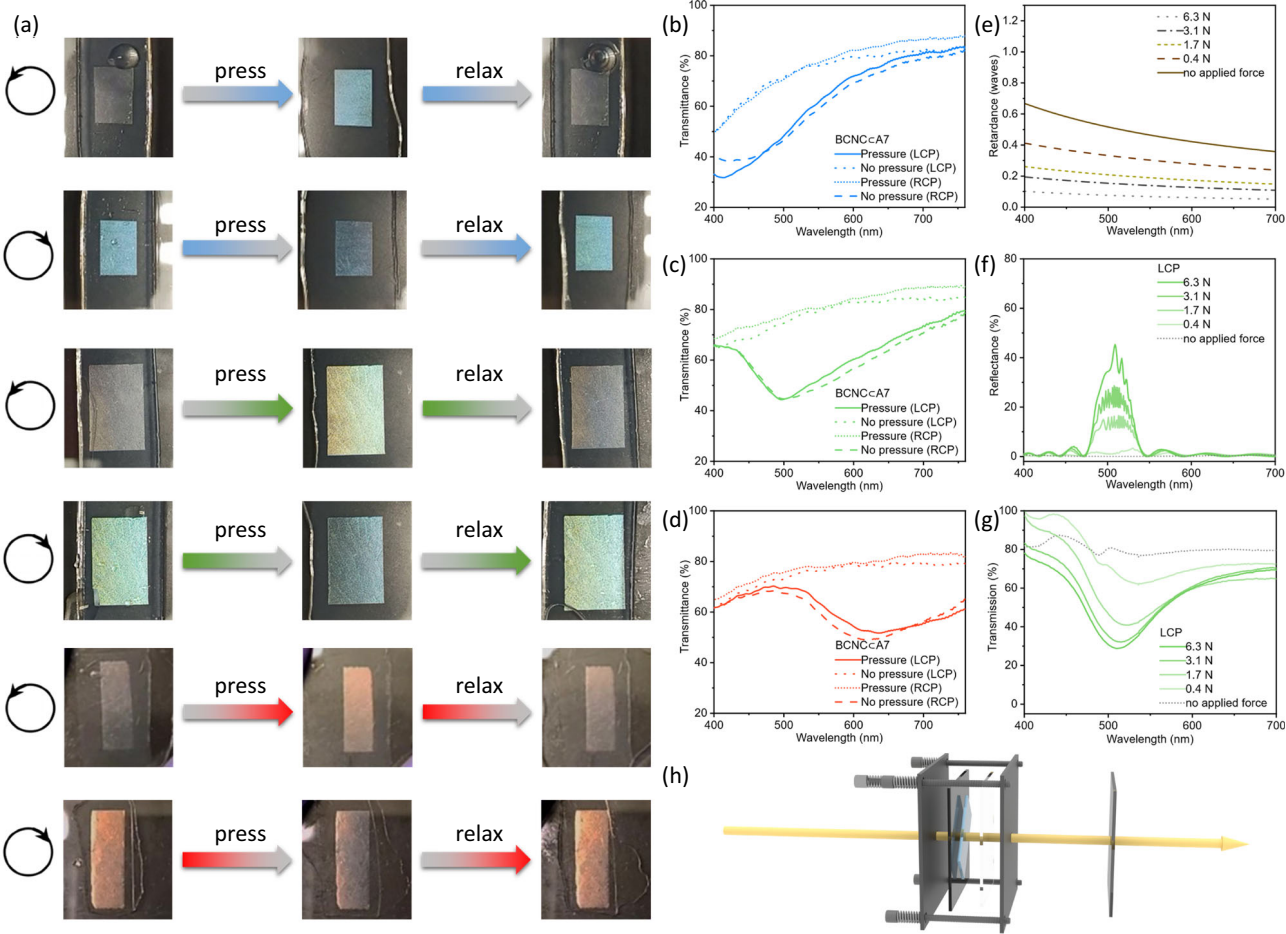
Optical response of chiral nematic CNC films combined with unidirectional CNC hydrogels under mechanical stress. **a** Photographs of chiral nematic CNC films with A7, showing color changes under applied pressure (about 5.0 ± 0.5 N for BCNC⊂A7, 5.1 ± 0.7 N for GCNC⊂A7 and 4.5 ± 0.2 N for RCNC⊂A7 films) and relaxation, captured through LCP and RCP filters. L-CPL and R-CPL transmission spectra for (**b**) BCNC⊂A7, (**c**) GCNC⊂A7 and (**d**) RCNC⊂A7, recorded in uncompressed or compressed states. **e** Spectral retardance for A7 under different mechanical force. **f** Simulated L-CPL reflectance spectra using Berreman’s method. **g** L-CPL transmission spectra for GCNC⊂A7 under different mechanical force. **h** Schematic of the experimental setup for control the compressive forces. The polarizer can be linear polarizer, or circular polarizer depending on the measurement. The widths are ~0.8, 0.9 cm and 0.5 cm for BCNC, GCNC and RCNC films, respectively.

Another potential application of the unidirectional CNC hydrogel is the development of mechano-responsive invertible chiral optical systems. Compared to traditional acrylic- or glass-based waveplates, hydrogel materials can be more elastic and this may allow mechanical forces to modulate the dimensions of hydrogels to influence the phase shift introduced by the unidirectional CNC hydrogels, thereby producing responsive chiral reflection colors. To investigate this potential, in our initial trial, one side of the A3 sample (this hydrogel sample was mounted on a glass slide) was first brought into contact with a separate glass slide that covered the chiral nematic CNC films. Subsequently, the entire A3 sample was pressed against this covering slide to apply pressure. However, despite this applied pressure, we did not observe obvious color changes recognizable through a circular polarizer. This lack of response is likely due to the thinness of A3, which made it difficult to induce changes in hydrogel dimensions in response to applied pressure. We thus increased the thickness and adjusted the particle concentration to make the unidirectional hydrogel A7, which also introduces a phase shift of ~ 0.33 to 0.65 waves across the visible spectrum. Similar to A3, placing A7 on top of chiral nematic CNC films with RGB color also results in the inversion of the chirality of the reflected light, as detected using a circular polarizer. When pressed, the visible R chiral reflection fades while the L chiral reflection becomes apparent; this L reflection can revert to its R reflection state by relaxation (Fig. 6a, Supplementary Fig. 9, Supplementary Fig. 10, Supplementary Movies 1 and 2). Importantly, this invertible chiral optical phenomenon controllable by pressure can be achieved for CNC films with different reflection colors over the visible spectrum. The cycling performance of this invertible chiral optical system enabled by mechanical forces was assessed by the compression-relaxation-compression cycling test shown in Supplementary Fig. 11. During the repeated cycles, the hydrogel remained intact, and the system showed repeated on/off color switching under circular polarizers. We also observed that after several cycles, the hydrogel became less elastic; elasticity could be recovered upon rehydration. These observations demonstrate the potential of switching between L and R chiral reflections enabled by mechanical force using sustainable materials.

The mechano-responsive, invertible chiral optical phenomena could be associated with changes in optical retardance due to variation in thickness or structural order. Direct observation of A7 under compression with parallel polarizers revealed that the hydrogel becomes transparent, similar to the appearance of A1 under parallel polarizers (A1 is a thin hydrogel that cannot alter the handedness of reflected light from chiral nematic CNC films), likely due to a reduction in thickness after compression that reduces the hydrogel’s retardance (Supplementary Fig. 12). 2D-XRD measurements were performed to assess structural changes after compression. The 2D-XRD pattern of freeze-dried A7 prepared in the presence of a compressive force of ~4.5 N remained anisotropic, but showed a slight reduction in structural order (S decreases from 0.38 to 0.31, Supplementary Fig. 4 and Supplementary Fig. 13). This suggests that the unidirectional structure is preserved after compression, but the degree of anisotropy decreases. Because orientational birefringence is generally related to the degree of structural anisotropy, this reduction is expected to lower the birefringence and, consequently, the retardance^37^. The reduced anisotropy may result from off-axis deformation (e.g., strain components not parallel to the CNC alignment) during compression. Because the material thickness also decreases under compression, and retardance scales with thickness, we assume that the reduced thickness, coupled with the decrease in structural order, may jointly lower the retardance, thereby modulating the optical properties of the chiral system. To investigate this, we built a custom setup in which screws and springs control the mechanical forces, allowing us to study the system’s optical properties under varying compressive forces (Fig. 6h). As the compressive load increased, the wave retardance decreased, and fell below 0.25 waves throughout the visible when the applied force exceeded ~3.1 N (Fig. 6e). The Δn under compression (Supplementary Fig. 14) did not change much when the applied force exceeded ~1.7 N, indicating that the reduction in thickness plays the dominant role in tuning the optical response at higher pressures. This tunability implies the possibility of using unidirectional materials to control the optical properties of chiral systems by applying pressure. The simulated left-handed circularly polarized reflection spectra predict a progressive decrease in reflectance with increasing load (Fig. 6f). Consistent with this trend, left-handed circularly polarized transmission spectra of chiral nematic CNC films in the presence of A7 shows a reduction in transmitted intensity with increasing force (Fig. 6g).

## Discussion

In summary, we developed a unidirectional CNC hydrogel that acts as a retardation layer with the ability to introduce a phase shift of ~0.33 to 0.65 waves over a broad range of visible wavelengths. Macroscopic observation of chiral nematic CNC films with the unidirectional CNC hydrogels on the top under white light shows the visible R chiral reflections, with the L chiral reflections being significantly suppressed. By taking advantage of the thickness changes and perturbation of the structural order under pressure, switching between L and R chiral reflections enabled by mechanical forces is realized. These inverted chiral optical phenomena could help guide the construction of stimuli-responsive cellulose-based devices for optical encryption or sensors, and has the potential to be extended to other materials systems such as rubber, resin, and more.

## Methods

### General

All chemicals and solvents were purchased from Sigma Aldrich unless otherwise indicated. Cellulose nanocrystal (CNC) suspensions in water were obtained from two different commercial sources, FPInnovations (3 wt%, pH ~6.6) and CelluForce (6.4 wt%, pH ~6.6). Clay samples were obtained from Kunimine Industries Co. Ltd. The morphology and structure of the samples were analyzed using a Zeiss CrossBeam 350 CryoFIB scanning electron microscope. Samples were sputter-coated with a 6 nm gold layer prior to imaging. Reflectance spectra were measured using an Ocean Optics setup comprising a FLAME-S-XR1 spectrometer, a DH2000-BAL tungsten-halogen light source, and an R400-7-UV-VIS reflection probe (all from Ocean Optics Inc., USA). Data collection and export were performed using OceanView software (v1.6.7). Samples were homogenized with a Thermo Scientific LP Vortex Mixer or a Durasonix 3 Litre Ultrasonic Cleaner (120 W, 40 kHz). Pressure can be measured using a Tekscan FlexiForce HT201 sensor, with resistance recorded under applied pressure. Polarized optical microscopy (POM) images were obtained using an Olympus BX53M polarizing optical microscope. Ultraviolet-visible (UV-vis) spectroscopy was performed on a UV-vis-NIR Cary 5000 spectrophotometer. Circular dichroism (CD) spectroscopy measurements were carried out using a JASCO J-815 spectrophotometer. To record the transmission and CD spectra for CNC samples with hydrogel in compressed states, the hydrogel was mounted on one glass slide and then pressed against another. It was thus sandwiched between two glass slides; its intrinsic stickiness preserved the compressed state throughout the measurements. Two-dimensional X-ray diffraction (2D-XRD) patterns were recorded using an APEX DUO diffractometer (Bruker) equipped with an APEX II charge-coupled device detector. Measurements were performed in transmission mode using a Cu Kα1 X-ray beam (λ = 0.154 nm) at 1 mA. Samples for 2D-XRD analysis were prepared by freezing the hydrogel either without compression or under an applied compressive force of ~4.5 N, followed by freeze-drying. Mechanical properties were assessed using a 5969 Series Universal Testing System (Instron) equipped with a 2 kN load cell under the compression mode. For each test, A7 was freshly prepared and cut into 1 × 1 cm^2^ squares. Real-time force and displacement were recorded at room temperature under the compression rate of 1 mm/min. The strain and stress were then calculated based on the original parameters (thickness and effective area) of the gel to obtain its characteristic stress-strain curve (Supplementary Fig. 15). The Young’s modulus of the gel was calculated from the initial linear portion of the stress-strain curves through linear regression analysis. Rheological properties of gels were characterized using an MCR 502 rheometer (Anton Paar) equipped with a parallel plate geometry (PP25/TG, diameter 25 mm) with an effective gap of 0.25 mm. All measurements were conducted at 20 °C under the strain-controlled and oscillatory shear modes. Frequency sweeps were performed over the frequency range from 0.1 to 100 rad/s with a 5% strain amplitude.

For quantification of the compressive force applied to hydrogel for spectral measurements, we used a custom setup in which screws and springs (METALLIXITY compression springs, spring rate, 0.59 N/mm) control the mechanical forces shown in Fig. 6h. The sample was held by either one polarizer and one glass plate, or two glass plates, depending on the measurement, and the entire stack was sandwiched between two polylactic acid plates with a hole (diameter, ~6 mm) at the center as a window for optical measurements. Compressive force can be quantified based on a change in the length of the springs from the initial position, measured using digital callipers by tightening the screws.

### Preparation of the unidirectional CNC hydrogel

Shear force and photopolymerization were used to form and lock-in the uniaxial alignment of CNCs and clay nanosheets to produce unidirectional hydrogels. For instance, to make hydrogel A3, a precursor for the hydrogels was prepared by mixing the 5.1 wt% CNC dispersion (4.0 g) with clay nanosheets (~ 40 mg), acrylamide (196 mg, 2.76 mmol), N,N’-methylenebisacrylamide (10 mg, 0.065 mmol) and 2-hydroxy-4’-(2-hydroxyethoxy)−2-methylpropiophenone (3 mg, 0.01 mmol). The hydrogel precursor was then transferred to a homemade setup (Supplementary Fig. 1) for shear alignment. The spacers were made by stacking adhesive tape to a thickness of ~300 µm for A1–A6 and ~550 µm for A7. A microscope slide was pressed down on top of the hydrogel precursor to shear the mixture, sliding it back and forth along the long axis for about 3 min at a rate of ~1.25 cm/s. Ultraviolet irradiation (300 nm UV-B lamp, 8 W) was then applied for 30 min to finish the photopolymerization. The obtained hydrogel was stored in water. The hydrogel heterogeneity was roughly assessed by comparing the retardance at 500 nm measured in the top, center, and bottom regions of the sample (Supplementary Fig. 16).

### Preparation of chiral nematic CNC films with visible colors

CNC films with chiral nematic structures were prepared by casting CNC suspensions into Petri dishes, followed by evaporation at room temperature. The CNC suspensions for casting were prepared by blending cellulose nanocrystal suspensions from two different sources, FPInnovations (3 wt%) and CelluForce (6.4 wt%). To prepare red CNC films, 2 mL of FPInnovations CNC suspension was mixed with 0.2 mL of CelluForce CNC suspension. The mixture was homogenized by shaking and sonication before being cast into Petri dishes and left to evaporate. To prepare green and blue CNC films, the proportions of the CNC suspensions were adjusted. For green CNC films, 1.2 mL of FPInnovations CNC suspension (3 wt%) was mixed with 0.6 mL of CelluForce CNC suspension (6.4 wt%). For blue CNC films, 1 mL of FPInnovations CNC suspension (3 wt%) was combined with 0.7 mL of CelluForce CNC suspension (6.4 wt%). All mixtures were homogenized in the same way and allowed to dry under identical conditions.

### Encapsulation of unidirectional CNC hydrogel pattern within a disordered hydrogel matrix

The unidirectional CNC hydrogel patterns can be fabricated by cutting a bulk hydrogel into the desired shapes or piecing small hydrogel strips together. The precursor solution for forming the disordered hydrogel matrix was composed of water, acrylamide, N,N′-methylenebisacrylamide, and 2-hydroxy-4′-(2-hydroxyethoxy)−2-methylpropiophenone, with a mass ratio of 10000:800:40:4, respectively. The hydrogel pattern was then immersed in this precursor solution within a silicone isolator on a glass slide. The assembly was subjected to ultraviolet (UV) irradiation for 1 h, leading to the successful encapsulation of the unidirectional CNC hydrogel pattern within the disordered hydrogel matrix.

## Supplementary information

Supplementary Information

Peer Review file

Description of Additional Supplementary Files

Supplementary Movie 1

Supplementary Movie 2

## Source data

Source Data

## Data Availability

The data that support the findings of this study are available within the article, Supplementary Information File, source data file or from the corresponding author upon request. Source data are provided with this paper.

## Code availability

The code used for simulation of optical properties is available in the Supplementary Information File or at https://github.com/supernaturerules/Berreman4x4_uniaxial_8c.
