# Supplementary material for: Unidirectional cellulose nanocrystal hydrogel for bio-based invertible chiral optics and sensors
Source: Nat Commun. 2026 Jun 3;17:7141. doi: 10.1038/s41467-026-73859-7 (PMC13396452; doi:10.1038/s41467-026-73859-7)
Supplement: Supplementary file 1 — Supplementary Information [file 41467_2026_73859_MOESM1_ESM.pdf]

Supplementary Information

Unidirectional Cellulose Nanocrystal Hydrogel for Bio-based Invertible Chiral Optics and Sensors

Yi-Tao Xu[1], Zongzhe Li[1], M. Andrea Ortiz Medrano[1], Mark J. MacLachlan[1,2,3,4]*

1. Department of Chemistry, University of British Columbia, 2036 Main Mall, Vancouver, British Columbia, V6T 1Z1, Canada

2. Stewart Blusson Quantum Matter Institute, University of British Columbia, 2355 East Mall, Vancouver, British Columbia, V6T 1Z4, Canada

3. WPI Nano Life Science Institute, Kanazawa University, Kanazawa 920-1192, Japan

4. Bioproducts Institute, University of British Columbia, 2360 East Mall, Vancouver, British Columbia, V6T 1Z3, Canada

** mmaclach@chem.ubc.ca*

1. Supplementary Discussion

1.1 Effect of retarder on the circularly polarized light

For left-handed circularly polarized light traveling in the z-direction (z=0), the electric field components in the x and y directions can be described as:

$E_x = E_0 \cos(\omega t)$

$E_y = E_0 \sin(\omega t)$

When a phase shift φ is introduced between the x and y components of the electric field, the wave can now be represented by:

$E_x = E_0 \cos(\omega t)$

$E_y = E_0 \sin(\omega t + \varphi)$

When $\varphi = \pi/2$, the light will become linearly polarized.

when $\varphi = \pi$, the light transitions to right-handed circularly polarized light.

when $\pi/2 < \varphi < 3\pi/2$, and $\varphi \neq \pi$ the light exhibits right-handed elliptical polarization.

2. Supplementary Methods

2.1 Retardance and birefringence as a function of wavelengths

For estimating the retardance and birefringence experimentally, the hydrogel was inserted between two parallel or crossed linear polarizers, with the shear direction oriented with a relative angle of 45° with respect to the transmission axes of the polarizers. In this situation, the transmission output is given by

$$T_{\parallel} = \cos^2(\varphi/2), \quad T_{\perp} = \sin^2(\varphi/2) \quad (1)$$

Here, φ denotes the wave-plate retardance, and can also be described by

$$\varphi = 2\pi d\Delta n/\lambda \quad (2)$$

where λ is the wavelength, d is the thickness of the birefringent material layer (in this case the hydrogel), and $\Delta n = |n_e - n_o|$ is the material birefringence, being n_o and n_e the ordinary and extraordinary refractive indices, respectively.

Because of the trigonometric functions, the experimental result will be an oscillating curve, and generates data in the range between 0 and 180°.

The retardance and birefringence curve is continuous according to Eq (2). For a continuous curve to read the accumulated phase retardance, Cauchy approximation, with normalized transmission data, can be introduced to estimate the retardance and birefringence for transparent, weakly dispersive birefringent materials:

$$\varphi = a/\lambda + b/\lambda^3 \quad (3)$$

$$T_{\parallel} = I_{\parallel} / (I_{\parallel} + I_{\perp}), \quad T_{\perp} = I_{\perp} / (I_{\parallel} + I_{\perp}) \quad (4)$$

Where a and b are constant parameters.[1, 2]

From the combination of equations (1) and (2), retardance in equations (5) and (6) can also be obtained. k is a non-negative integer that describes the order of the solution, and it is possible to manually choose k for each set of data (Supplementary Fig. 17).[2]

$$\varphi = k\pi + 2\arctan\sqrt{\frac{T_\perp}{T_\parallel}} \quad (5)$$

$$\varphi = (k+1)\pi - 2\arctan\sqrt{\frac{T_\perp}{T_\parallel}} \quad (6)$$

2.2 Optical simulations

Simulations were performed based on the Berreman 4 × 4 matrix method.[3] Code for simulation was written in Python3 language based on the open-source frameworks developed by Olivier Castany[4] and Gen Kamita[5]. A uniaxial layer was inserted between the environment (isotropic half space, air) and the chiral nematic stacks. Note that we did not know the n_o and n_e for the uniaxial layer, however, since Δn can be obtained from Cauchy approximation, we manually set $n_o = 1.3$, $n_e = 1.3 + \Delta n$. The values of chiral nematic pitch were estimated from the UV–vis spectra. Sample thickness was measured using digital calipers. The adapted Python script (incorporating the retardation layer into Kamita's implementation) is provided as follows: (.py file is available at https://github.com/supernaturerules/Berreman4x4_uniaxial_8c)

```
"""
Berreman4x4_uniaxial_8c
====
Note
  Simulation of chiral nematic materials in the presence of a uniaxial retardation layer by the Berreman4x4 matrix method.
  Berreman4x4.py is required for using this module.
  Codes are developed based on the previous simulation framework developed by Olivier Castany and Gen Kamita.
Yitao Xu/Berreman4x4_uniaxial_8c: Optical Simulation of Cholesteric Liquid Crystals with an Overlying uniaxial Layer/supernature.rules@gmail.com
"""
```

```
import Berreman4x4
from numpy import sin, abs, array, linspace, append, arange, savetxt
from Berreman4x4 import pi, e_y
from matplotlib import pyplot
from time import localtime
from collections import namedtuple
from copy import deepcopy


class Simulator(object):
    """


    Properties
      pitch:        pitch in nm (180 degree twist)
      no:           refractive index that the ordinary ray experiences
      ne:           refractive index that the extraordinary ray experiences
      nAverage:     Average refractive index, that is (no + ne)/2
      nDelta:       Birefringence, that is ne - no
      nSuperstrate: refractive index of superstrate (air: n = 1)
      nSubstrate:   refractive index of suberstrate (PS: n = 1.59, SiO2: n=1.55)
      stack:        number of pitches (180 degree twist) in the structure
```

```
  angle:        angle of incidence in degrees
  lbda_min:     wavelength min (in meters)
  lbda_max:     wavelength max (in meters)
  points:       number of data points in simulation
  slices:       number of discrete anisotropic layers per 180 twist
  c:            thickness of the top uniaxial layer (nm)
  a:            coefficient for retardance dispersion law (a/lbda)
  b:            coefficient for retardance dispersion law (b/lbda^3)

Attribute
  settings:     All parameters related to the above properties are stored here.
"""

def __init__(self):
    # Added 'c', 'a', 'b' to settings
    self.settings = dict(no=1.524, ne=1.586, nSubstrate=1.59, nSuperstrate=1, pitch=0, stack=0, angle=0,
                         lbda_min=400,
                         lbda_max=800, points=101, slices=23,
                         c=0, a=0, b=0)

def prepare(self):
    sup = Berreman4x4.IsotropicNonDispersiveMaterial(self.settings['nSuperstrate'])
    sub = Berreman4x4.IsotropicNonDispersiveMaterial(self.settings['nSubstrate'])
```

```
front = Berreman4x4.IsotropicHalfSpace(sup)

back = Berreman4x4.IsotropicHalfSpace(sub)


# --- Base Chiral Stack Setup ---

LC = Berreman4x4.UniaxialNonDispersiveMaterial(self.settings['no'], self.settings['ne'])  # ne along
z

R = Berreman4x4.rotation_v_theta(e_y, pi / 2)  # rotation of pi/2 along y

LC = LC.rotated(R)  # apply rotation from z to x


# Cholesteric pitch:

# One half turn of a left-handed helix. To change to right-hand, angle=+pi.

TN = Berreman4x4.TwistedMaterial(LC, self.settings['pitch'], angle=-pi, div=self.settings['slices'])


# Inhomogeneous layer, repeated layer (The Stack)

IL = Berreman4x4.InhomogeneousLayer(TN)

L_stack = Berreman4x4.RepeatedLayers([IL], self.settings['stack'])


# --- Wavelength Loop ---

lbda_list = wavelength(self.settings)

J_list = []



for lbda in lbda_list:
```

```
k0 = 2 * pi / lbda

# Calculate dispersion for the top layer
# Law: (a / lbda + b / lbda^3) / 2 / pi

a = self.settings.get('a', 0)
b = self.settings.get('b', 0)
c = self.settings.get('c', 0)

# Avoid division by zero
if lbda == 0: lbda = 1e-9

# Calculate retardance/delta_n term
ret_val = (a / lbda + b / (lbda ** 3))
delta_n_top = ret_val * lbda / (2 * pi * c)

#Create the Top Uniaxial Layer
# no = 1.3, ne = 1.3 + delta_n
no_top = 1.3
ne_top = 1.3 + delta_n_top

TopMat = Berreman4x4.UniaxialNonDispersiveMaterial(no_top, ne_top)
```

```
# Rotate top layer to be in-plane (ne along x) similar to the LC start
# If you need a specific azimuth for the retarder, rotate around Z here.
TopMat = TopMat.rotated(Berreman4x4.rotation_v_theta(Berreman4x4.e_y, pi / 2))


# Build Structure


layer_list = [L_stack]
if c > 0:
    # Assuming HomogeneousLayer exists for a slab of constant material



    try:
        TopLayer = Berreman4x4.HomogeneousLayer(TopMat, c, hs_method="Padé", hs_order=3)
    except AttributeError:


        TopFlat = Berreman4x4.TwistedMaterial(TopMat, c, angle=0, div=1)
        TopLayer = Berreman4x4.InhomogeneousLayer(TopFlat)




    layer_list.insert(0, TopLayer)


s = Berreman4x4.Structure(front, layer_list, back)
```

```
        # Calculate Jones Matrix for this wavelength
        J_now = s.getJones(sin(self.settings['angle'] * pi / 180), k0)
        J_list.append(J_now)

    J = array(J_list)

    # Jones matrices for the circular wave basis
    Jc = Berreman4x4.circularJones(J)
    power_c = abs(Jc) ** 2
    return power_c

def calculate(self):
    power_c = self.prepare()
    # Right-circular wave is reflected in the stop-band.
    # R_LR, T_LR close to zero.
    # r = Spectrum(Berreman4x4.extractCoefficient(power_c, 'r_RR'), self.settings,
    # type='right circularly polarised light')
    # l = Spectrum(Berreman4x4.extractCoefficient(power_c, 'r_LL'), self.settings)
    r = Spectrum(Berreman4x4.extractCoefficient(power_c, 't_pp'), self.settings,
                type='right circularly polarised light')
    l = Spectrum(Berreman4x4.extractCoefficient(power_c, 't_ss'), self.settings)

    LandR = namedtuple('LandR', 'l r')
```

```
    result = LandR(l, r)
    return result

def calculateR(self):
    power_c = self.prepare()
    # Right-circular wave is reflected in the stop-band.
    # R_LR, T_LR close to zero.
    spectrum = Spectrum(Berreman4x4.extractCoefficient(power_c, 'r_RR'), self.settings, type='r_left')
    return spectrum

def calculateL(self):
    power_c = self.prepare()
    # Left-circular wave is transmitted in the full spectrum.
    # T_RL, R_RL, R_LL close to zero, T_LL close to 1.
    spectrum = Spectrum(Berreman4x4.extractCoefficient(power_c, 'r_LL'), self.settings)
    # transmission = Spectrum(1 - reflection.spectrum, self.settings, type='t_left')
    # r_and_t = namedtuple('r_and_t', 'r t')
    # spectra = r_and_t(reflection, transmission)
    return spectrum

def matrix(self, parameter, start, stop, step=1):
    """
    Returns batch-simulated spectra.
```

```
parameter: String that specifis which parameter to vary.
"""
leftArray = array([])
if parameter in self.settings:

    for x in arange(start, stop, step):
        self.settings[parameter] = x
        result = self.calculateL()
        leftArray = append(leftArray, result.spectrum)

elif parameter == 'nDelta':

    for x in arange(start, stop, step):
        self.nDelta = x
        result = self.calculateL()
        leftArray = append(leftArray, result.spectrum)

elif parameter == 'nAverage':

    for x in arange(start, stop, step):
        self.nAverage = x
        result = self.calculateL()
        leftArray = append(leftArray, result.spectrum)
```

```
        else:
            return 0
        leftMatrix = leftArray.reshape(-1, self.settings['points'])
        Result = Spectrum(leftMatrix, self.settings)
        return Result


    @property
    def nDelta(self):
        return self.settings['ne'] - self.settings['no']

    @nDelta.setter
    def nDelta(self, nDelta):
        half = nDelta / 2.0
        nAverage = self.nAverage
        self.settings['no'] = nAverage - half
        self.settings['ne'] = nAverage + half

    @property
    def nAverage(self):
        return (self.settings['no'] + self.settings['ne']) / 2.0
```

```
@nAverage.setter
def nAverage(self, nAverage):
    nDelta = self.nDelta
    self.settings['no'] = nAverage - nDelta / 2.0
    self.settings['ne'] = nAverage + nDelta / 2.0

@property
def no(self):
    return self.settings['no']

@no.setter
def no(self, no):
    self.settings['no'] = no

@property
def ne(self):
    return self.settings['ne']

@ne.setter
def ne(self, ne):
    self.settings['ne'] = ne

@property
```

```
def pitch(self):
    return self.settings['pitch']

@pitch.setter
def pitch(self, pitch):
    self.settings['pitch'] = pitch

@property
def nSuperstrate(self):
    return self.settings['nSuperstrate']

@nSuperstrate.setter
def nSuperstrate(self, nSuperstrate):
    self.settings['nSuperstrate'] = nSuperstrate

@property
def nSubstrate(self):
    return self.settings['nSubstrate']

@nSubstrate.setter
def nSubstrate(self, nSubstrate):
    self.settings['nSubstrate'] = nSubstrate
```

```
@property
def stack(self):
    return self.settings['stack']

@stack.setter
def stack(self, stack):
    self.settings['stack'] = stack

@property
def angle(self):
    return self.settings['angle']

@angle.setter
def angle(self, angle):
    self.settings['angle'] = angle

@property
def lbda_min(self):
    return self.settings['lbda_min']

@lbda_min.setter
def lbda_min(self, lbda_min):
    self.settings['lbda_min'] = lbda_min
```

```
@property
def lbda_max(self):
    return self.settings['lbda_max']

@lbda_max.setter
def lbda_max(self, lbda_max):
    self.settings['lbda_max'] = lbda_max

@property
def points(self):
    return self.settings['points']

@points.setter
def points(self, points):
    self.settings['points'] = points

@property
def slices(self):
    return self.settings['slices']

@slices.setter
def slices(self, slices):
```

```
        self.settings['slices'] = slices

    @property
    def c(self):
        return self.settings['c']

    @c.setter
    def c(self, c):
        self.settings['c'] = c

    @property
    def a(self):
        return self.settings['a']

    @a.setter
    def a(self, a):
        self.settings['a'] = a

    @property
    def b(self):
        return self.settings['b']

    @b.setter
```

```
    def b(self, b):
        self.settings['b'] = b


class Spectrum(object):
    """
    Output of Simulator.

    Method
      plot: Plots the spectrum.
      save: Exports the spectrum as a txt file in the current directory.
            It will also save the settings and time it was saved in the header.

    Attribute
      spectrum: The simulated spectrum.
      settings: The setting which was used for the simulation are stored here.
    """

    def __init__(self, spectrum, settings, type='r_left'):
        self.spectrum = spectrum
        self.settings = deepcopy(settings)
        self.settings['type'] = type
```

```
def plot(self):
    fig = pyplot.figure()
    ax = fig.add_subplot(111)
    lbda_list = wavelength(self.settings)
    ax.plot(lbda_list, self.spectrum, '--', label=self.settings['type'])
    ax.set_xlabel(r"Wavelength $\lambda_0$ (nm)")
    ax.set_ylabel(r"Reflectance $R$")
    fmt = ax.xaxis.get_major_formatter()
    fmt.set_powerlimits((-3, 3))
    pyplot.show()

def image(self):
    lbda_list = wavelength(self.settings)
    fig = pyplot.imshow(self.spectrum, extent=[self.settings['lbda_min'], self.settings['lbda_max'], 90, 0])
    pyplot.xlabel(r"Wavelength [nm]")
    pyplot.ylabel(r"Angle [degrees]")
    pyplot.colorbar()
    pyplot.show()


def save(self, filename='spectrum.txt'):
    self.settings['savedtime'] = localtime()
    try:
```

```
            savetxt(filename, self.spectrum, header=str(self.settings))
        except TypeError:
            savetxt(filename, self.spectrum)
            print ("Header not included in the output text due to the version of your numpy module.")


def wavelength(settings):
    """
    Used for calculating wavelength.
    :param settings: Dictionary "B4x4.settings"
    :return: ndarray of wavelength
    """
    return linspace(settings['lbda_min'], settings['lbda_max'], settings['points'])


if __name__ == '__main__':
    mySim = Simulator()
    # Example usage with new layer:
    # mySim.c = 1000  # thickness in nm
    # mySim.a = 500    # dummy dispersion Coeff
    mySpec = mySim.calculateL()
    mySpec.plot()
```

3. Supplementary Tables

Supplementary Table 1 Composition of hydrogel precursors

Sample	CNC (mg/mL)	clay (mg/mL)	monomer (mg/mL)	cross-linker (mg/mL)	initiator (mg/mL)	Spacer thickness (μm)
**A1**	45	13	50	2.5	0.75	300
**A2**	49	12	50	2.5	0.75	300
**A3**	51	11	50	2.5	0.75	300
**A4**	52	11	50	2.5	0.75	300
**A5**	55	10	50	2.5	0.75	300
**A6**	57	10	50	2.5	0.75	300
**A7**	49	11	48	2	0.75	550

4. Supplementary Figures

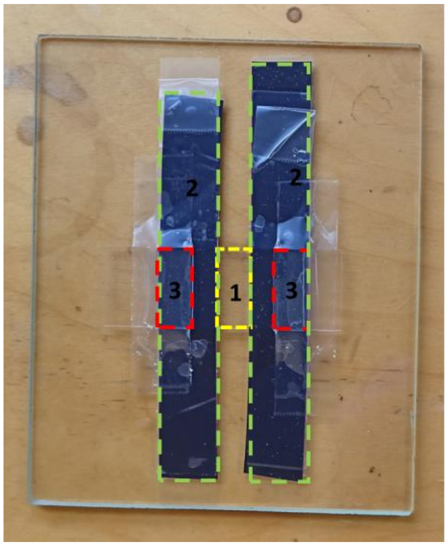

1. Drop-casting area
2. Spacers created using sticky tape
3. Glass pieces for securing the position of the capping slide during shearing

Supplementary Fig. 1 Custom experimental setup for shearing hydrogel precursors. The length of the yellow (or red) rectangular outline is ~ 25 mm.

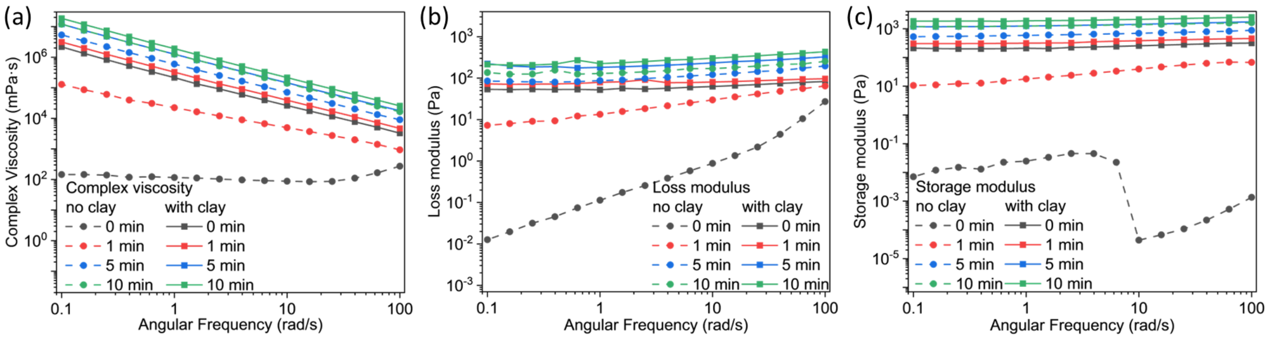

Supplementary Fig. 2 Rheological properties of A3 with and without clay nanosheets during polymerization. Frequency-dependent (a) complex viscosity, (b) loss modulus and (c) storage modulus for A3 (■ with straight line) and A3 without clay nanosheets (● with dash line) before and during polymerization.

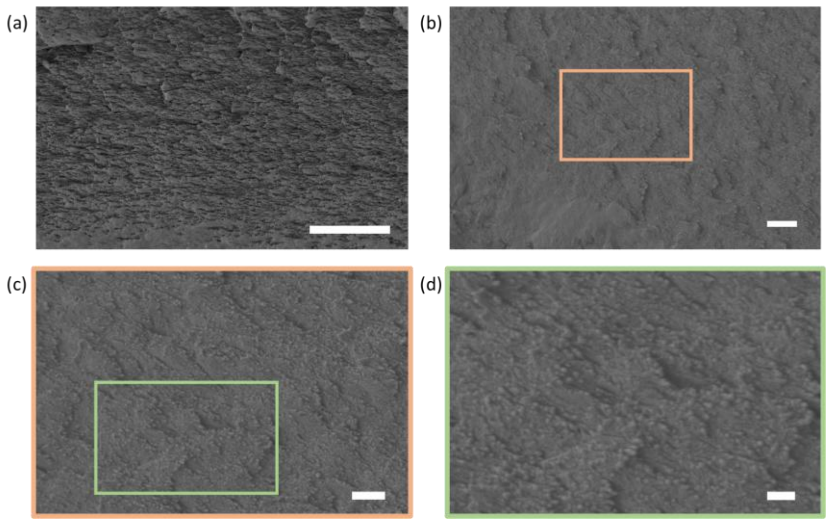

Supplementary Fig. 3 Cross-sectional SEM images of A3 hydrogel. SEM images were acquired at different magnifications. Scale bars: (a) 5 µm, (b) 1 µm, (c) 500 nm, and (d) 200 nm.

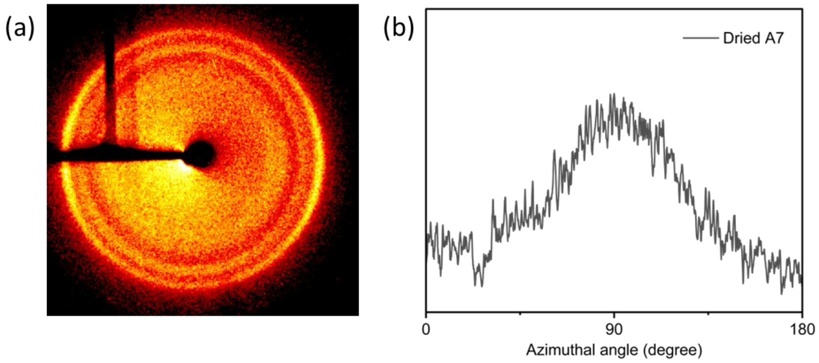

Supplementary Fig. 4 X-ray diffraction analysis of alignment of CNCs in the A7 hydrogel. Two-dimensional (2D) XRD pattern (a) and corresponding azimuthal-dependent intensity distributions (b) for A7. Hermans order parameter (S) was ~ 0.38 and calculated according to previous work using the following equations[6]:

$$S = \frac{3\langle\cos^2\gamma\rangle - 1}{2} \tag{7}$$

$$\langle\cos^2\gamma\rangle = 1 - 2\langle\cos^2\emptyset\rangle \tag{8}$$

$$\langle\cos^2\emptyset\rangle = \frac{\int I(\emptyset)\cos^2\emptyset\sin\emptyset\, d\emptyset}{\int I(\emptyset)\sin\emptyset d\emptyset} \tag{9}$$

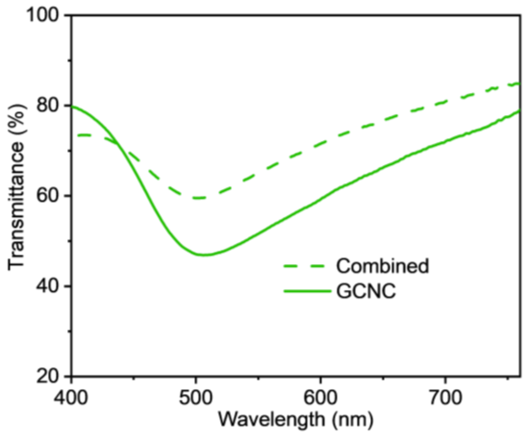

Supplementary Fig. 5 Optical properties of GCNC and GCNC⊂A6. Transmission spectra of the bare green cellulose nanocrystal film (GCNC) compared with the combined L-CPL and R-CPL transmission spectra of GCNC⊂A6. The transmittance of the combined transmission spectrum is calculated as the average of $T_{GCNC⊂A6}$ (RCP) and $T_{GCNC⊂A6}$ (LCP), given by:

$$T_{\mathrm{combined}} = \frac{T_{\mathbf{GCNC\subset A6}}\ (\mathrm{RCP}) + T_{\mathbf{GCNC\subset A6}}\ (\mathrm{LCP})}{2} \qquad (10)$$

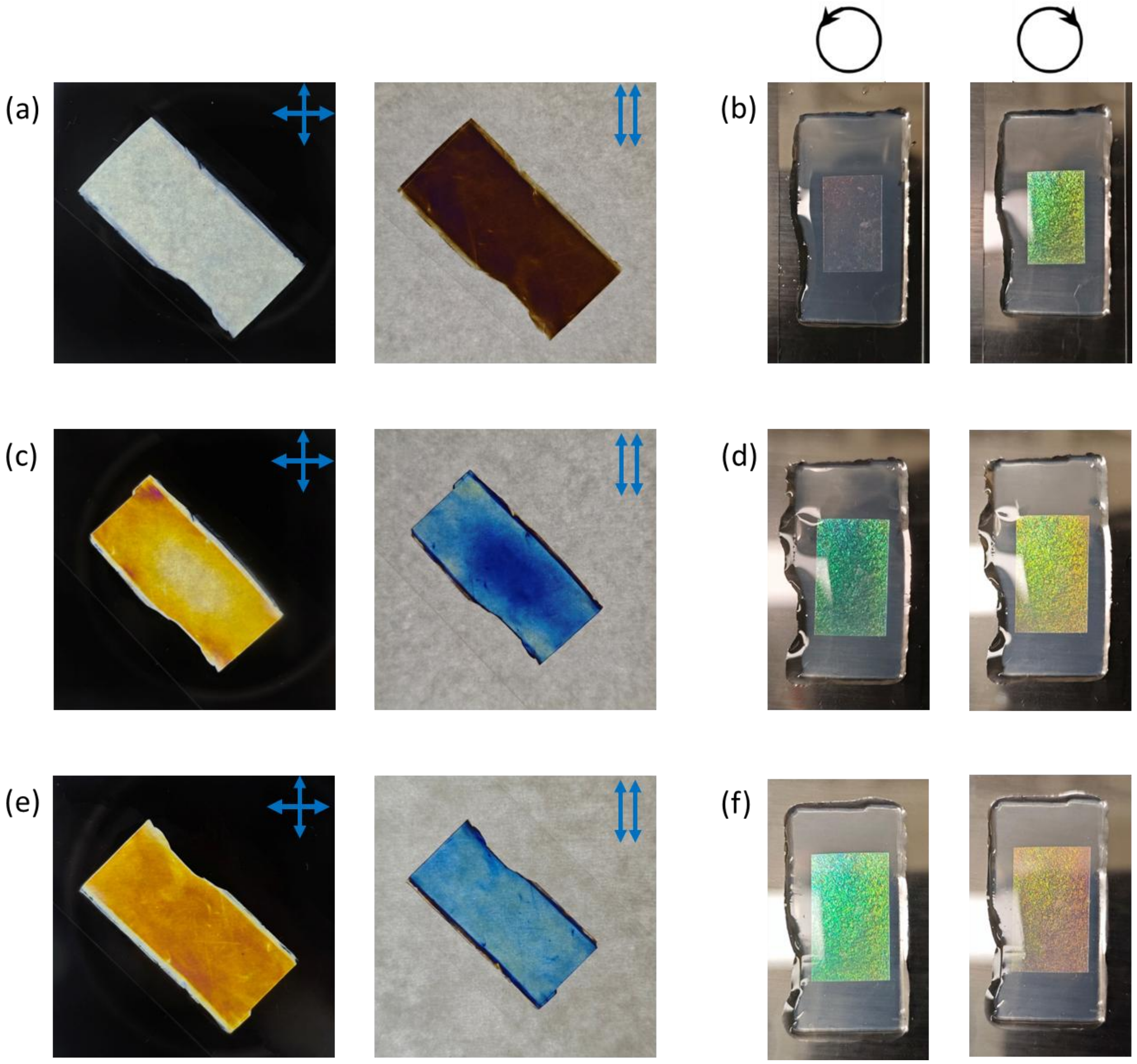

Supplementary Fig. 6 Optical response of A7 and GCNC⊂A7 to water evaporation and ionic strength. (a,c,e) Transmission images of hydrogel A7 recorded under crossed polarizers (left) and parallel polarizers (right): (a) as prepared (no water evaporation), (c) after water evaporation at room temperature for 2.5 h, and (e) after treatment with 1 mM NaCl solution. (b,d,f) Photographs of the chiral nematic CNC films combined with the corresponding unidirectional CNC hydrogels shown in (a,c,e). Photographs in (b,d,f) were acquired through left-handed circular polarizer (left) and right-handed circular polarizer (right). The length of the hydrogel is ~ 2.3 cm.

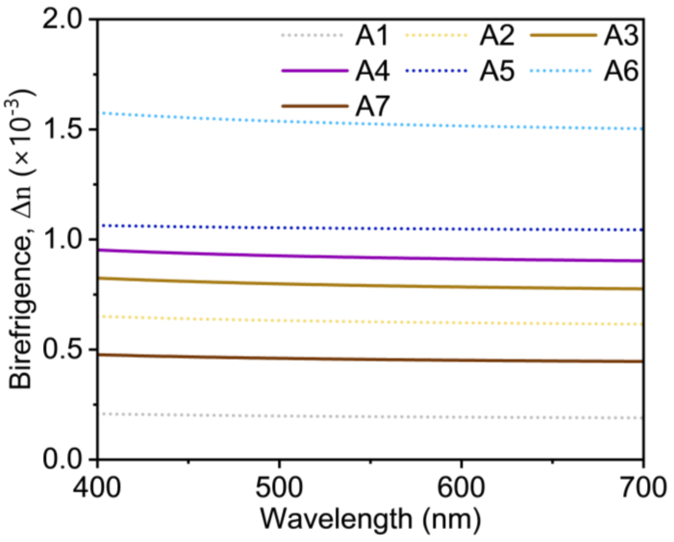

Supplementary Fig. 7 The birefringence dispersions for A1-A7 from 400 – 700 nm. The birefringence was estimated based on Cauchy approximation.

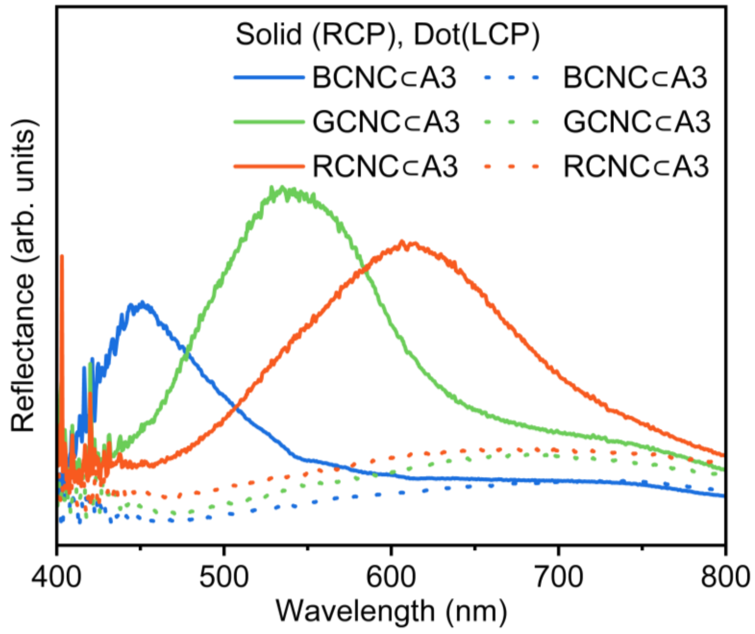

Supplementary Fig. 8 Reflectance spectra of materials. L-CPL (dot) and R-CPL (solid) reflectance spectra for BCNC⊂A3, GCNC⊂A3 and RCNC⊂A3.

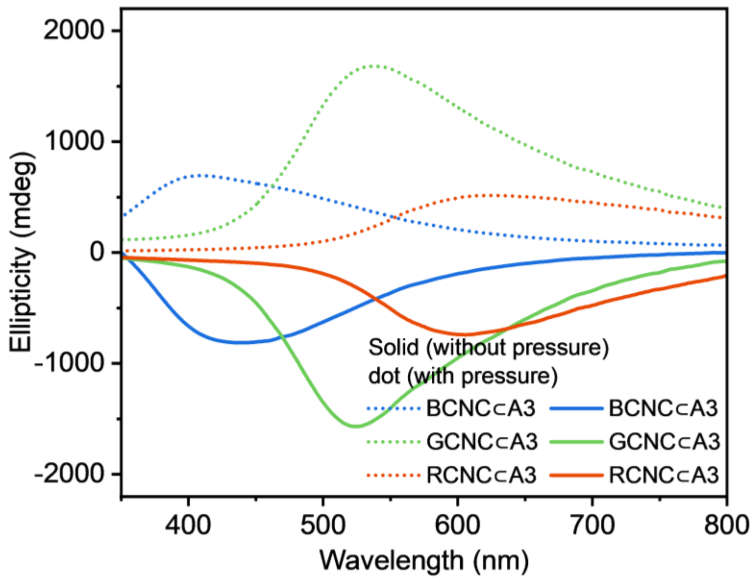

Supplementary Fig. 9 CD spectra of materials. CD spectra of BCNC⊂A3, GCNC⊂A3 and RCNC⊂A3 without (solid line) and with applied pressure (dotted line).

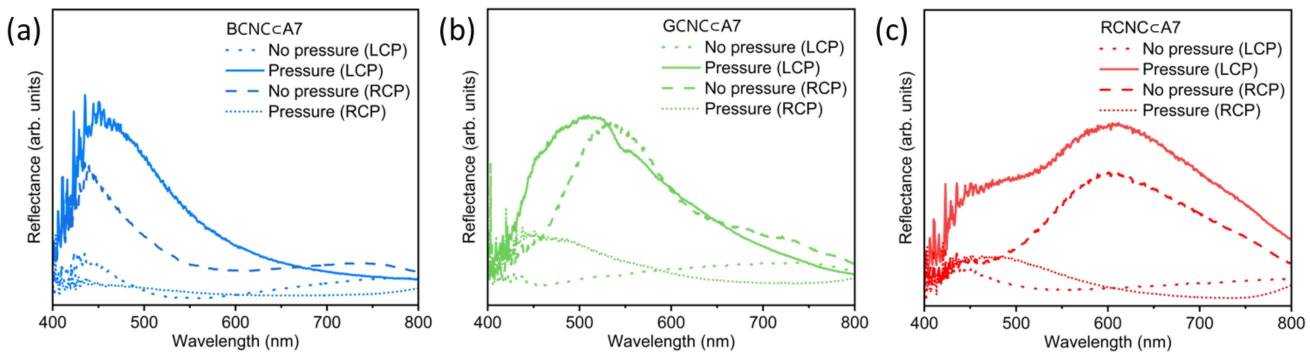

Supplementary Fig. 10 Reflectance spectra of materials. L-CPL and R-CPL reflectance spectra for BCNC⊂A7 (a), GCNC⊂A7 (b) and RCNC⊂A7 (c), recorded in uncompressed or compressed states.

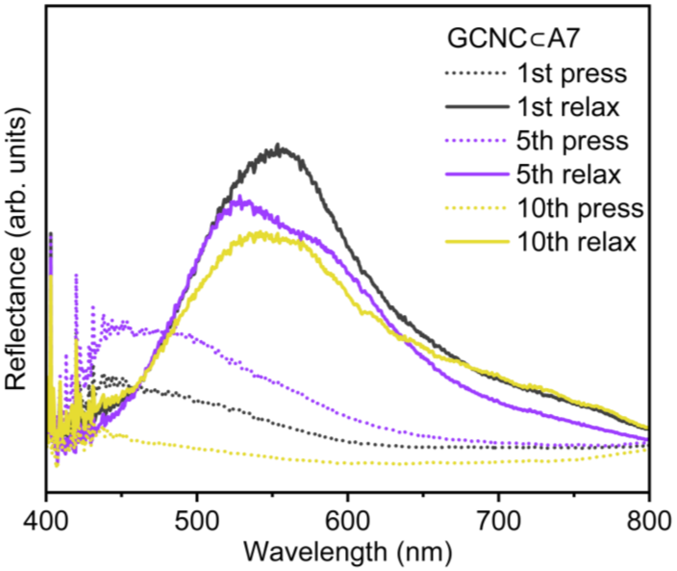

Supplementary Fig. 11 Compression-relaxation-compression cycling tests. Relectance spectra for GCNC⊂A7 following one, five, and ten cycles of pressing and relaxing.

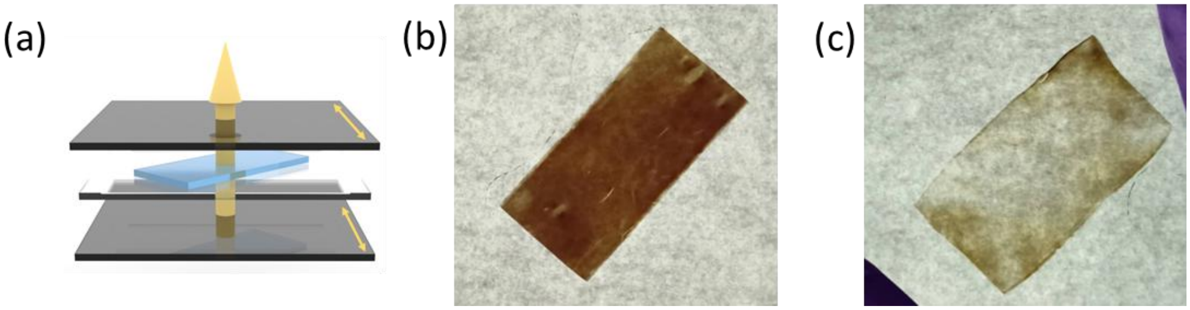

Supplementary Fig. 12 Pressure-dependent optical response of A7 under parallel polarizers. (a) Schematic of the setup used to observe A7 under parallel polarizers. (b, c) Photographs of A7 without (b) and with pressing (c) under parallel polarizers. The shear direction is oriented at 45º relative to the polarization axis of either polarizer. Upon pressing, the hydrogel's ability to block incident linearly polarized light is diminished, likely due to alterations in the phase shift caused by the reduction in thickness and the disruption of the unidirectional structure. The length of the hydrogel sample is ~ 2.3 cm.

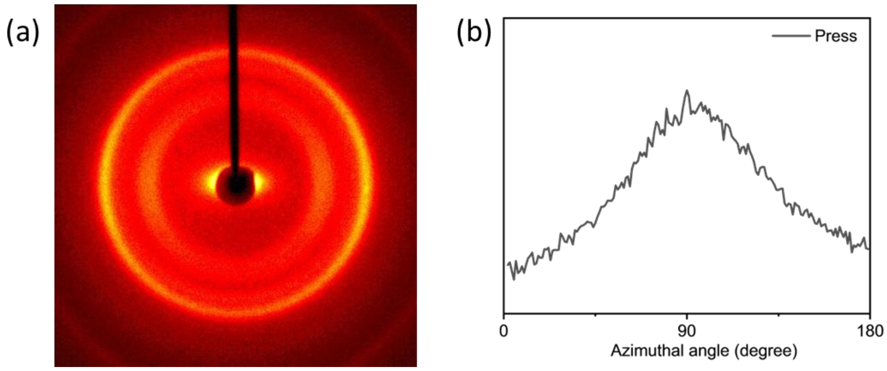

Supplementary Fig. 13 X-ray analysis of the alignment of CNCs in A7. Two-dimensional (2D) XRD pattern (a) and corresponding azimuthal-dependent intensity distributions (b) for freeze-dried A7 under pressure.

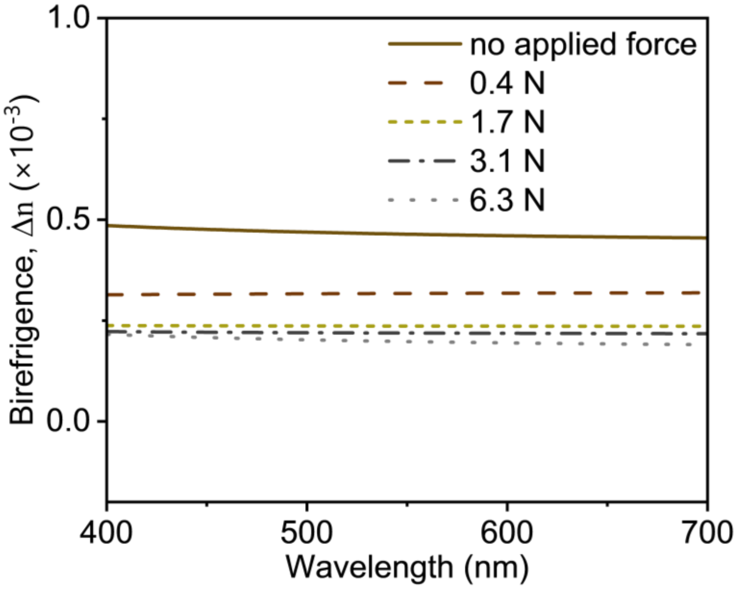

Supplementary Fig. 14 Birefringence dispersions of A7 under different compressive forces. The birefringence was estimated by fitting the Cauchy formula.

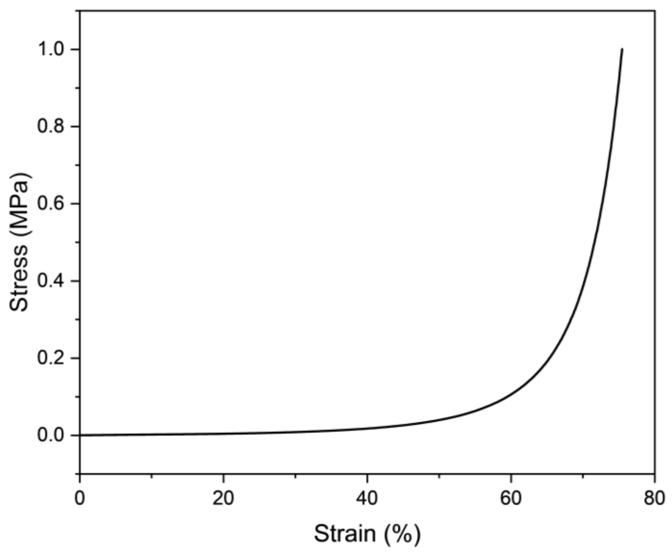

Supplementary Fig. 15 Stress-strain curve of A7. The compressive modulus was measured to be 21 kPa.

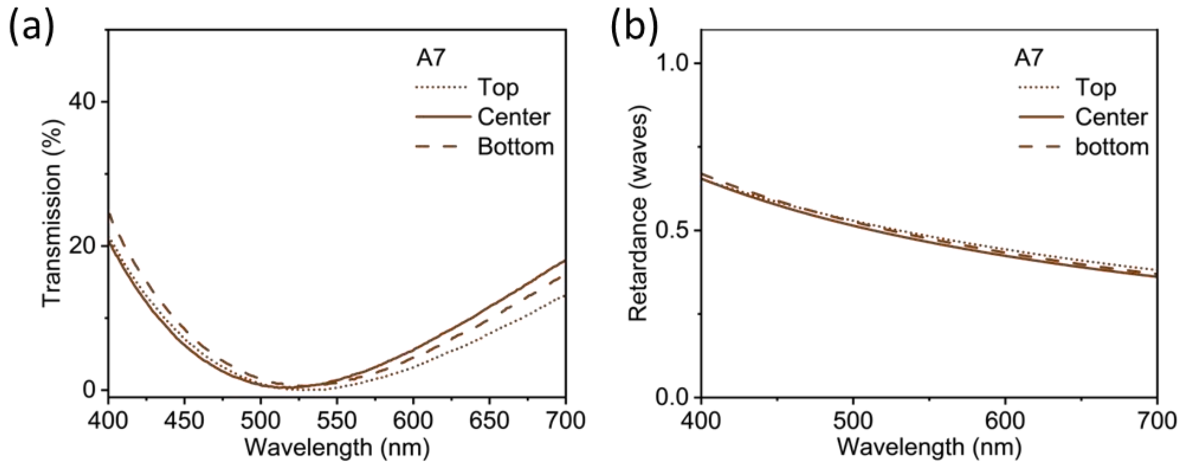

Supplementary Fig. 16 Hydrogel heterogeneity estimation. (a) Transmission spectra measured under parallel polarizers for the top, center, and bottom regions of sample A7. (b) Corresponding spectral retardance for the same regions of sample A7.

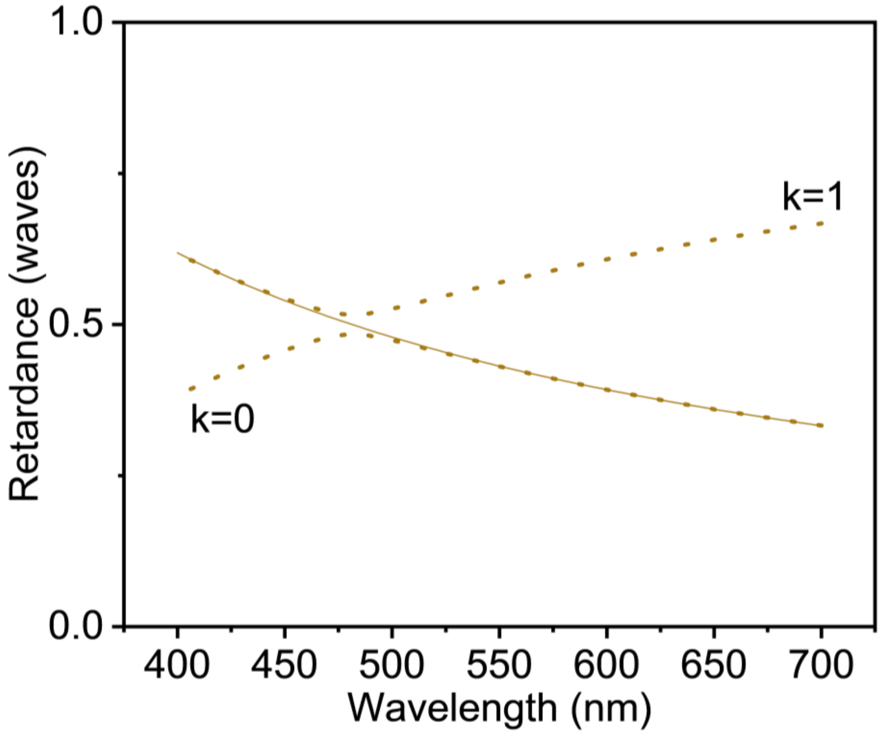

Supplementary Fig. 17 Calculated birefringence for A3. The birefringence dispersions for A3 calculated using equations (5), (6) with the indicated constants k and curves fitted using Cauchy formula, equation (3).

5. Supplementary References

1. Messaadi, A., Sánchez-López, M. d. M., García-Martínez, P., Vargas, A., Moreno, I. Optical system for measuring the spectral retardance function in an extended range. *J. Eur. Opt. Soc.-Rapid Publ.* **12**, 21 (2016).
2. Bhupathi, P., *et al.* Optical birefringence in uniaxially compressed aerogels. *New J. Phys.* **12**, 103016 (2010).
3. Berreman, D. W. Optics in Stratified and Anisotropic Media: 4×4-Matrix Formulation. *J. Opt. Soc. Am. A* **62**, 502-510 (1972).
4. Castany, O. Berreman4x4/Berreman4x4: Python implementation of Berreman's 4x4 matrix method. GitHub (2012). Available at: https://github.com/Berreman4x4/Berreman4x4. (Accessed: 28th September 2025).
5. Kamita, G. Gkamita/B4X4: Simple optical simulation of cholesteric liquid crystals. GitHub (2016). Available at: https://github.com/gkamita/B4x4. (Accessed: 28th September 2025)
6. Kose, O., Boott, C. E., Hamad, W. Y., MacLachlan, M. J. Stimuli-Responsive Anisotropic Materials Based on Unidirectional Organization of Cellulose Nanocrystals in an Elastomer. *Macromolecules* **52**, 5317-5324 (2019).
