# Supplementary material for: Unidirectional cellulose nanocrystal hydrogel for bio-based invertible chiral optics and sensors
Source: Nat Commun. 2026 Jun 3;17:7141. doi: 10.1038/s41467-026-73859-7 (PMC13396452; doi:10.1038/s41467-026-73859-7)
Supplement: Supplementary file 3 — Description of Additional Supplementary Files [file 41467_2026_73859_MOESM3_ESM.pdf]

Supplementary Movie 1: Pressure-driven switching between left- and right-handed chiral reflections in CNC films displaying blue, green, and red structural colors, enabled by a unidirectional CNC hydrogel. Video recorded under a left-handed circular polarizer.

Supplementary Movie 2: Pressure-driven switching between left- and right-handed chiral reflections in CNC films displaying blue, green, and red structural colors, enabled by a unidirectional CNC hydrogel. Video recorded under a right-handed circular polarizer.
