# Supplementary material for: Unidirectional cellulose nanocrystal hydrogel for bio-based invertible chiral optics and sensors
Source: Nat Commun. 2026 Jun 3;17:7141. doi: 10.1038/s41467-026-73859-7 (PMC13396452; doi:10.1038/s41467-026-73859-7)
Supplement: Supplementary file 2 — Peer Review file [file 41467_2026_73859_MOESM2_ESM.pdf]

nature portfolio

Peer Review File

Unidirectional Cellulose Nanocrystal Hydrogel for Bio-based Invertible Chiral Optics and Sensors

Corresponding Author: Professor Mark MacLachlan

This file contains all reviewer reports in order by version, followed by all author rebuttals in order by version.

Version 0:

Reviewer comments:

Reviewer #1

(Remarks to the Author)
In their manuscript, Xu and colleagues deliver an elegant demonstration of a unidirectionally aligned cellulose-nanocrystal (CNC) hydrogel that acts as a broadband (≈0.3–0.5 λ) retardation layer, flipping the handedness of circularly polarised reflections from underlying chiral-nematic CNC films and permitting that inversion to be toggled on and off with modest mechanical pressure. The writing is well to follow and the experiments are logical; yet the clarity of presentation also exposes two weak points—how far this concept advances beyond existing wave-plate approaches and how securely its physical mechanism is established. Let me detail these points below.

Although the authors position their hydrogel as the first CNC-based medium to give broadband right-handed reflection, the Introduction itself recounts several precedents in which nematic or birefringent interlayers (thermotropic, photoresponsive or cellulose-based) suppressed left-handed reflection or generated dual-handed output, inspired by Chrysina resplendens and related biological systems. What distinguishes the present study—hydrogel elasticity and pressure tunability—should therefore be quantified head-to-head: bandwidth of inversion, magnitude and cycling stability of the pressure stimulus, environmental robustness, and sustainability benefits. This is missing.

The mechanistic explanation of the observed rotations rest chiefly on two qualitative observations: compression thins the hydrogel (reducing phase delay $\varphi = 2\pi\Delta n\, d / \lambda$) and disrupts its uniaxial order, as signalled by an isotropic 2D-XRD pattern under load. Yet thickness is discussed only in passing (“thin A3 showed no effect”) and pressure appears as single-point values (~4.5–5 N) without error bars or cycling data. A systematic map of retardance versus controlled strain would let the simple Jones-matrix picture be tested quantitatively together with EM images; the birefringence Δn could be tracked through polarimetry to separate geometric from structural changes. Such data would either validate or falsify the current hypothesis and would immediately inform the “designer materials” ambition of this paper and leave me without a doubt in the interpretation.

Closely related, the composition-space explored (A1–A7) already hints that clay content, monomer fraction and UV dose tune both modulus and retardance: only A3, A4 and A7 sit in the desired 0.4–0.5 λ window across the visible spectrum. Explaining why the other formulations miss the target, and whether that failure stems from Δn, thickness or polydomain defects, would turn an empirical recipe into a design rule. Even a rudimentary finite-element model—elastic compression coupled to optical phase delay—could outline the parameter ranges needed for specific colours or switching thresholds. Or a mechanistic approach to a “phase map” of the effect of each of the ingredients.

It would be worthwhile to give the scalability and reproducibility more space in the manuscript, e.g.,: How uniform is retardance over centimetre areas? Does repeated wetting or drying alter Δn? Are pressures of a few newtons sufficient for devices thicker than the laboratory samples?

Furthermore, some text in the figures is too small to read and/or cropped. Please correct.

Reviewer #2

(Remarks to the Author)
This work focuses on controlling the handedness of reflected light by enabling switching between left- and right-handed

chiral reflections using CNC-based materials. A CNC hydrogel was developed and employed as a responsive retardation layer, enabling the conversion of left-handed chiral reflections from chiral CNC nematic films into right-handed chiral reflections. The optical properties of this bio-based unidirectional structure were found to be pressure-sensitive, which the authors attributed to thickness variations or disruptions in the alignment of the birefringent layer.

The formulation of A6 and A5 is very similar; the main difference is the quantity of CNCs (55 mg/mL for A5 and 57 mg/mL for A6). Can the authors explain why A6 is so selective ("…only alters the handedness of certain wavelengths of light (i.e., light in the orange and red spectral region") compared, for example, with A5?

Why were clay nanosheets used to increase the viscosity and not a polymer, or is the role of the clay other than increasing the viscosity? If the clays were not used, would the hydrogels present the same behaviour?
For the preparation of the unidirectional CNC hydrogel "A microscope slide was pressed down on top of the hydrogel precursor to shear the mixture, sliding it back and forth along the long axis for about 3 mins." What was the value of the shear rate applied? Rheological measurements performed during both shear and cross-linking would be valuable to elucidate the role of the clay nanosheets further.
The results presented are interesting, and after minor revision, the manuscript should be accepted.

Reviewer #3

(Remarks to the Author)
The present study reports the development of CNC-based materials exhibiting right-handed reflections, achieved using CNC hydrogels with aligned CNCs that have been cross-linked. The alignment and the structural parameters of the hydrogels strongly influence the colors. The study is clearly written and easy to follow. Before publication I have the following suggestions of improvement.

Major:

1. I recommend that the authors move Table S1 (or parts of Table S1) into the main text for easier comparison of the samples. In addition, how do the differences between samples relate to their optical properties?

2. Regarding alignment through shearing, does this work uniformly regardless of sample thickness, or are there heterogeneities across the thickness?

3. The spectra in figure 2e and 2f should be simulated taken into account the optical characteristics of the hydrogel.

4. Since the retardation plate is a hydrogel, it should be prone to water loss. Does this happen? How would this affect its optical properties?

5. In Figure 6, how quickly do the materials relax back to regain their colors?

Minor:

1. The font sizes in the figures should be made uniform, please compare all figures. For example, in Figure 1(f), the x- and y-axis labels are very small and hard to read. In Figure 2(e), the y-axis text is unclear, as the letter "T" is not properly reproduced.

2. In the Experimental section, "Preparation of the unidirectional CNC hydrogel", how many times is the sliding performed, and what is the spacer distance? Please add more details to this section.

Version 1:

Reviewer comments:

Reviewer #1

(Remarks to the Author)
The authors have substantially revised the manuscript in response to my earlier comments - thank you!The optical characterization has been expanded and is internally consistent, with broadband handedness inversion demonstrated across blue, green, and red CNC films. These additions significantly strengthen the mechanistic clarity and credibility of the work, and the presentation is much improved relative to the original submission.

That said, the study remains largely phenomenological. While the retardance window required for handedness inversion is now clearly articulated, the manuscript stops short of delivering a rigorous structure–property framework. Direct structural evidence under relevant compressive conditions is limited, and comparisons to prior waveplate-based or interlayer approaches remain qualitative - and I would think that some of this is needed for a publication in this journal.

Reviewer #2

(Remarks to the Author)
The authors answered almost all the questions and comments raised by the referees, and the manuscript can be accepted.

Reviewer #3

(Remarks to the Author)
the authors have addressed all of my previous comments. I have no further comments to add.

Version 2:

Reviewer comments:

Reviewer #1

(Remarks to the Author)
Thank you for these last clarifications.

Open Access This Peer Review File is licensed under a Creative Commons Attribution 4.0 International License, which permits use, sharing, adaptation, distribution and reproduction in any medium or format, as long as you give appropriate credit to the original author(s) and the source, provide a link to the Creative Commons license, and indicate if changes were made.
In cases where reviewers are anonymous, credit should be given to 'Anonymous Referee' and the source.
The images or other third party material in this Peer Review File are included in the article's Creative Commons license, unless indicated otherwise in a credit line to the material. If material is not included in the article's Creative Commons license and your intended use is not permitted by statutory regulation or exceeds the permitted use, you will need to obtain permission directly from the copyright holder.
To view a copy of this license, visit https://creativecommons.org/licenses/by/4.0/

RESPONSE TO REVIEWERS

REVIEWER 1

In their manuscript, Xu and colleagues deliver an elegant demonstration of a unidirectionally aligned cellulose-nanocrystal (CNC) hydrogel that acts as a broadband (≈0.3–0.5 λ) retardation layer, flipping the handedness of circularly polarised reflections from underlying chiral-nematic CNC films and permitting that inversion to be toggled on and off with modest mechanical pressure. The writing is well to follow and the experiments are logical; yet the clarity of presentation also exposes two weak points—how far this concept advances beyond existing wave-plate approaches and how securely its physical mechanism is established. Let me detail these points below.

We thank the reviewer for the positive comments and suggestions for improvement. We have added additional results and addressed the concerns point by point as follows:

1. Although the authors position their hydrogel as the first CNC-based medium to give broadband right-handed reflection, the Introduction itself recounts several precedents in which nematic or birefringent interlayers (thermotropic, photoresponsive or cellulose-based) suppressed left-handed reflection or generated dual-handed output, inspired by Chrysina resplendens and related biological systems. What distinguishes the present study—hydrogel elasticity and pressure tunability—should therefore be quantified head-to-head: bandwidth of inversion, magnitude and cycling stability of the pressure stimulus, environmental robustness, and sustainability benefits. This is missing.

The materials and the optical properties shown in this work differ substantially from previously reported CNC-based chiral nematic optical systems. Earlier works (refs. 20–28) could generate right-handed reflection but either failed to eliminate left-handed reflection in CNC-based systems or did not produce visible colors. Furthermore, as the reviewer noted, another distinction of our design is that the presence of this retardation layer allows reversible switching between left- and right-handed reflections. We employed multiple optical characterization techniques—including circularly polarized light (CPL) transmission spectra (Figure 3e, 3f, 6b-d), CPL reflectance spectra (Figure S8, S10), CD spectra (Figure 3g and S9), and reflection circularly polarized optical microscopy (Figure 4)—to confirm these invertible optical properties. The results show that handedness inversion of reflected light can be achieved for CNC films with blue, green, and red structural colors, covering a wavelength range of approximately 400–680 nm.

Regarding the effect of compressive force on optical properties, our response is provided in the reply to Comment 2.

We also performed additional experiments to evaluate the cycling performance of this type of optical system. The hydrogel shows a compressive modulus of 21 kPa, typical of soft hydrogel materials. Scaling to thicker, stiffer, or more elastic materials appears feasible; however, we still need more exploration of the mechanical properties of unidirectionally aligned CNC hydrogels. In future work, we will explore strategies to broaden the accessible mechanical range, including the introduction of physical crosslinkers (e.g., polyvinylpyrrolidone, PVP) or reinforcing additives.

New results with descriptions are highlighted in the manuscript and provided here as well:

The cycling performance of this invertible chiral optical system enabled by mechanical forces was assessed by the compression-relaxation-compression cycling test shown in **Figure S11**. During the repeated cycles, the hydrogel remained intact, and the system showed repeated on/off color switching under circular polarizers. We also observed that after several cycles, the hydrogel became less elastic; elasticity could be recovered upon rehydration. (Page 17)

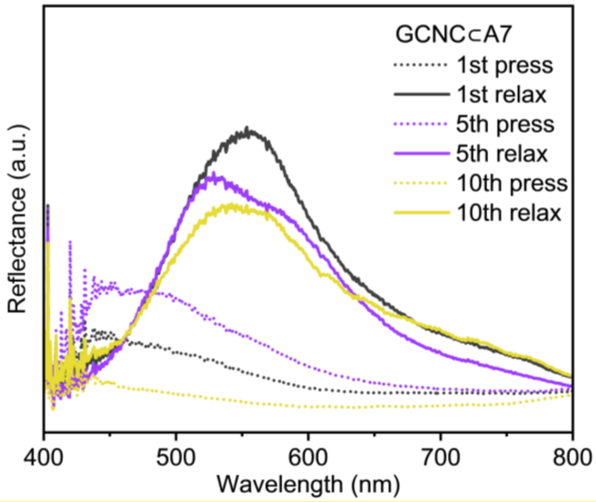

Figure S11. Compression-relaxation-compression cycling test for the **GCNC⊂A7**.

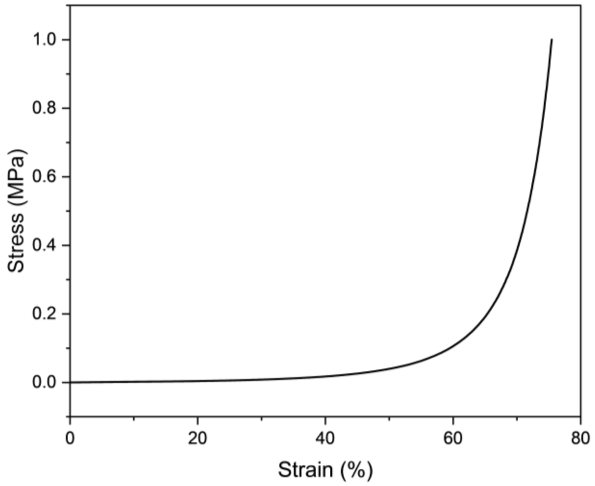

Figure S14. Stress-strain curve of **A7**. The compressive modulus was measured to be 21 kPa.

2. The mechanistic explanation of the observed rotations rest chiefly on two qualitative observations: compression thins the hydrogel (reducing phase delay $\varphi = 2\pi\Delta n\, d / \lambda$) and disrupts its uniaxial order, as signalled by an isotropic 2D-XRD pattern under load. Yet thickness is discussed only in passing ("thin A3 showed no effect") and pressure appears as single-point values (~4.5–5 N) without error bars or cycling data. A systematic map of retardance versus controlled strain would let the simple Jones-matrix picture be tested quantitatively together with EM images; the birefringence Δn could be tracked through polarimetry to separate geometric from structural changes. Such data would either validate or falsify the current hypothesis and would immediately

inform the "designer materials" ambition of this paper and leave me without a doubt in the interpretation.

We agree with the reviewer that it is important to clarify how applied pressure influences birefringence and reflectance. Accordingly, we performed additional experiments and simulations and provided error bars for Figure 6a. Based on these results, we conclude that pressure primarily modulates the optical response through changes in retardance arising from thickness variation, and we have revised the manuscript to emphasize this as the dominant mechanism. We have removed the 2D-XRD data acquired under pressure because we consider that it may not provide reliable support for the mechanism in the present context. In our setup, the measurements were conducted under higher pressures than those used in the optical experiments, and clear XRD signals could only be obtained in the dry state (the wet hydrogel yields showed insufficiently resolved patterns), and therefore, the 2D-XRD results acquired under pressure are not directly comparable to the conditions of interest.

The results and their corresponding descriptions are highlighted in the manuscript and are also summarized here: (Note that to generate a continuous retardance curve, we used a Cauchy approximation. Because a continuous retardance curve is used, the retardance window for chiral-reflection inversion is expressed as $\pi/2 < \varphi < 3\pi/2$. Details for the inversion of chiral reflection, obtaining the continuous retardance curve and for performing the simulations are provided in Section 1, Sections 2 and 3 of the Supplementary Information. For brevity and to avoid redundancy, we do not repeat the full methodology here)

The mechano-responsive, invertible chiral optical phenomena could be associated with changes in optical retardance due to thickness variation. Direct observation of **A7** under compression with parallel polarizers revealed that the hydrogel becomes transparent, similar to the appearance of **A1** under parallel polarizers (**A1** is a thin hydrogel that cannot alter the handedness of reflected light from chiral nematic CNC films), likely due to a reduction in thickness that reduces the hydrogel's retardance (**Figure S12**). To investigate this, we built a custom setup in which screws and springs control the mechanical forces, allowing us to study the system's optical properties under varying compressive forces (**Figure 6h**). As the compressive load increased, the wave retardance decreased, and fell below 0.25 waves throughout the visible spectrum when the applied force exceeded ~3.1 N (**Figure 6e**). The Δn under compression (**Figure S13**) did not change much when the applied force exceeded ~1.7 N, indicating that the reduction in thickness plays the dominant role in tuning the optical response at higher pressures. This tunability implies the possibility of using unidirectional materials to control the optical properties of chiral systems by applying pressure. The simulated left-handed circularly polarized reflection spectra predict a progressive decrease in reflectance with increasing load (**Figure 6f**). Consistent with this trend, left-handed circularly polarized transmission spectra of chiral nematic CNC films in the presence of **A7** show a reduction in transmitted intensity with increasing force (**Figure 6g**). (Page 18-19)

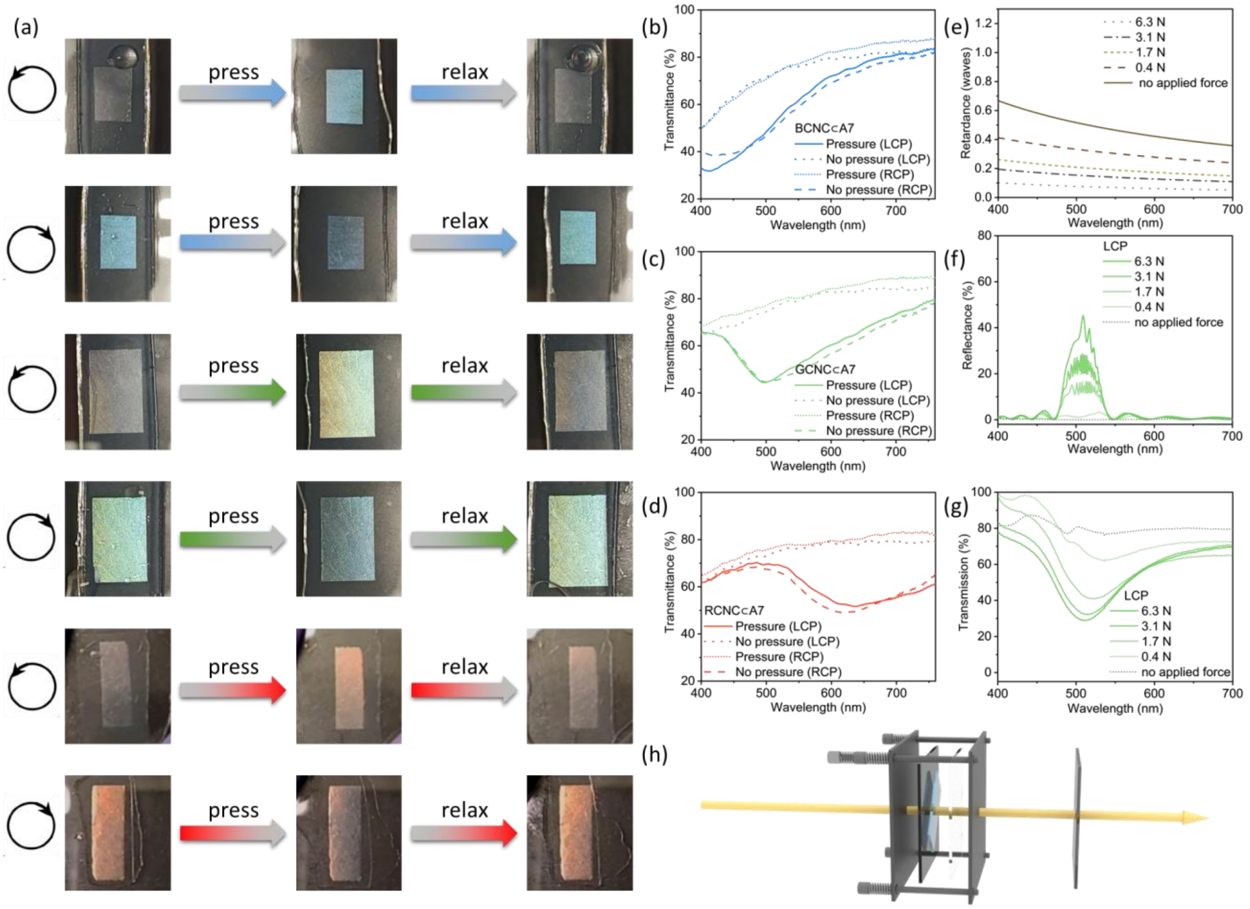

Figure 1. Optical response of chiral nematic CNC films combined with unidirectional CNC hydrogels under mechanical stress. (a) Photographs of chiral nematic CNC films with **A7**, showing color changes under applied pressure (about 5.0 ± 0.5 N for **BCNC⊂A7**, 5.1 ± 0.7 N for **GCNC⊂A7** and 4.5 ± 0.2 N for **RCNC⊂A7** films) and relaxation, captured through LCP and RCP filters. (b-d) L-CPL and R-CPL transmission spectra for (b) **BCNC⊂A7**, (c) **GCNC⊂A7** and (d) **RCNC⊂A7**, recorded in uncompressed or compressed states. (e) Spectral retardance for **A7** under different mechanical force. (f) Simulated L-CPL reflectance spectra using Berreman's method (g) L-CPL transmission spectra for **GCNC⊂A7** under different mechanical force. (h) Schematic of the experimental setup for control the compressive forces. The polarizer can be a linear polarizer, or circular polarizer depending on the measurement. The widths are ~ 0.8, 0.9 cm and 0.5 cm for **BCNC**, **GCNC** and **RCNC** films, respectively.

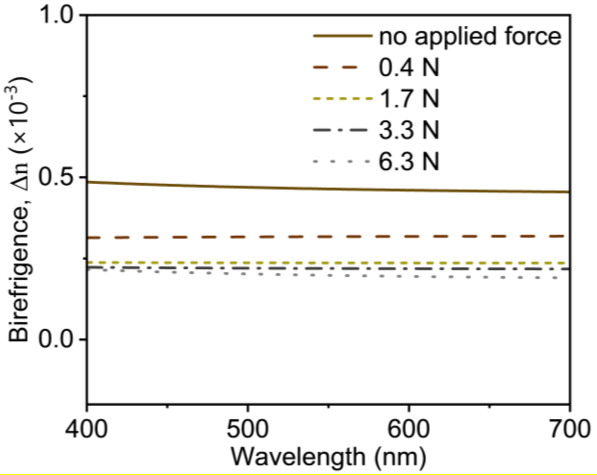

Figure S13. The birefringence dispersions of **A7** under different compressive forces.

3. Closely related, the composition-space explored (A1–A7) already hints that clay content, monomer fraction and UV dose tune both modulus and retardance: only A3, A4 and A7 sit in the desired 0.4–0.5 λ window across the visible spectrum. Explaining why the other formulations miss the target, and whether that failure stems from Δn, thickness or polydomain defects, would turn an empirical recipe into a design rule. Even a rudimentary finite-element model—elastic compression coupled to optical phase delay—could outline the parameter ranges needed for specific colours or switching thresholds. Or a mechanistic approach to a "phase map" of the effect of each of the ingredients.

The reviewer's comment about a design rule for unidirectional hydrogel fabrication is interesting and important. Developing a reliable rule, however, requires a large, systematically acquired dataset that isolates the effects of each component, sample thickness, and key processing parameters. We plan to pursue this using machine learning approach in future work. The initial thought is to link birefringent color to the underlying optical and compositional variables—including birefringence (Δn), thickness, component concentrations, and processing conditions—and to compile these relationships into a color map database suitable for machine-learning assisted prediction. Practically, the birefringent color will be recorded as digital images, yielding RGB values. These color metrics, together with measured thickness and known/estimated dispersion behavior, will give a color map database and provide substantial data for data training and prediction using suitable open-source algorithm. The goal is to enable forward and inverse predictions (e.g., mapping a target color/retardance to recommended precursor concentrations and processing windows). We intend to explore and implement this strategy in future studies.

Regarding the differences in optical properties, these can be attributed to variations in retardance. Additional results, and discussions have been added to describe the observed behaviors, as follows:

Interestingly, by carefully adjusting the particle concentration or hydrogel thickness, the unidirectional hydrogels (**A2**–**A5** and **A7**) can exhibit a uniform white transmission color (**Figure 1d**) when viewed between crossed polarizers (the hydrogel was placed at 45° between two crossed polarizers). The corresponding transmission spectra show a very broad peak spanning the visible region. These results suggest that these hydrogels can rotate the polarization of incident linearly

polarized light over a broad range of wavelengths, enabling light of various wavelengths within the visible spectrum to pass through the second polarizer (i.e., the analyzer, **Figure 1g**). Further increasing the total particle concentration gives hydrogels (**A6**) that show interference color at specific wavelengths, indicating that the polarization rotation of incident linearly polarized light occurs over relatively narrower spectral bands. (Page 6)

This phenomenon can be ascribed to the retardance differences of **A6** across the visible spectrum. In the orange and red spectral region (610-700 nm), the phase shift introduced by **A6** is > 0.25 waves, which results in hydrogel **A6** altering the handedness of light. In the blue–green region (400-560 nm), the phase shift is > 0.75 waves, so the reflection in this region remains left-handed and the reflection peak narrows, producing the distinct cyan color. The combined L and R circularly polarized reflection resembles that of the bare CNC film (**Figure S3**), supporting the above analysis that hydrogel **A6** only alters the handedness of certain wavelengths of light (i.e., light in the orange and red spectral region). As an example for comparison, hydrogel **A5** introduces a phase shift of ~ 0.44 to 0.75 waves over 425-700 nm, enabling the inversion of green chiral reflection from the underlying CNC film rather than producing light reflection of both left and right handedness. (Page 9)

To also visualize how the retardation layer with varying Δn affects the chiral reflection from the ideal chiral nematic stacks (e.g., without defects or pitch variation), we performed numeric simulation (see Section 3 in supplementary information for details) by means of the 4 × 4 Berreman matrix for a multilayer model (air ∣ uniaxial materials ∣ chiral nematic stacks ∣ silica substrate).[33-35] Note that n_0 and n_e for defining the uniaxial materials is unknown; however, since Δn can be derived from Cauchy approximation (**Figure S7**), we manually set $n_0 = 1.3$ and $n_e = 1.3+\Delta n$. The simulated spectra (**Figure 2g** and **2h**) predict that **A2**, **A3**, **A4** and **A5** effectively invert the chiral reflection from the underlying chiral nematic stacks with markedly increased right-handed reflectance and strongly suppressed left-handed components, whereas **A1** and **A6** do not invert the chiral reflection, in agreement with the experimental measurements. The simulated reflection bands are noticeably narrower than the experimental spectra. This can be ascribed to the CNC film's heterogeneities, including defect formation and pitch-length variations across the film thickness (e.g., due to drying dynamics, gravity-driven gradients or flow effects), which broaden the chiral nematic stop band and yield a wider peak in measurements (this broadening also contribute to the observed orange reflection under RCP for **GCNC⊂A6**). (Page 10-11)

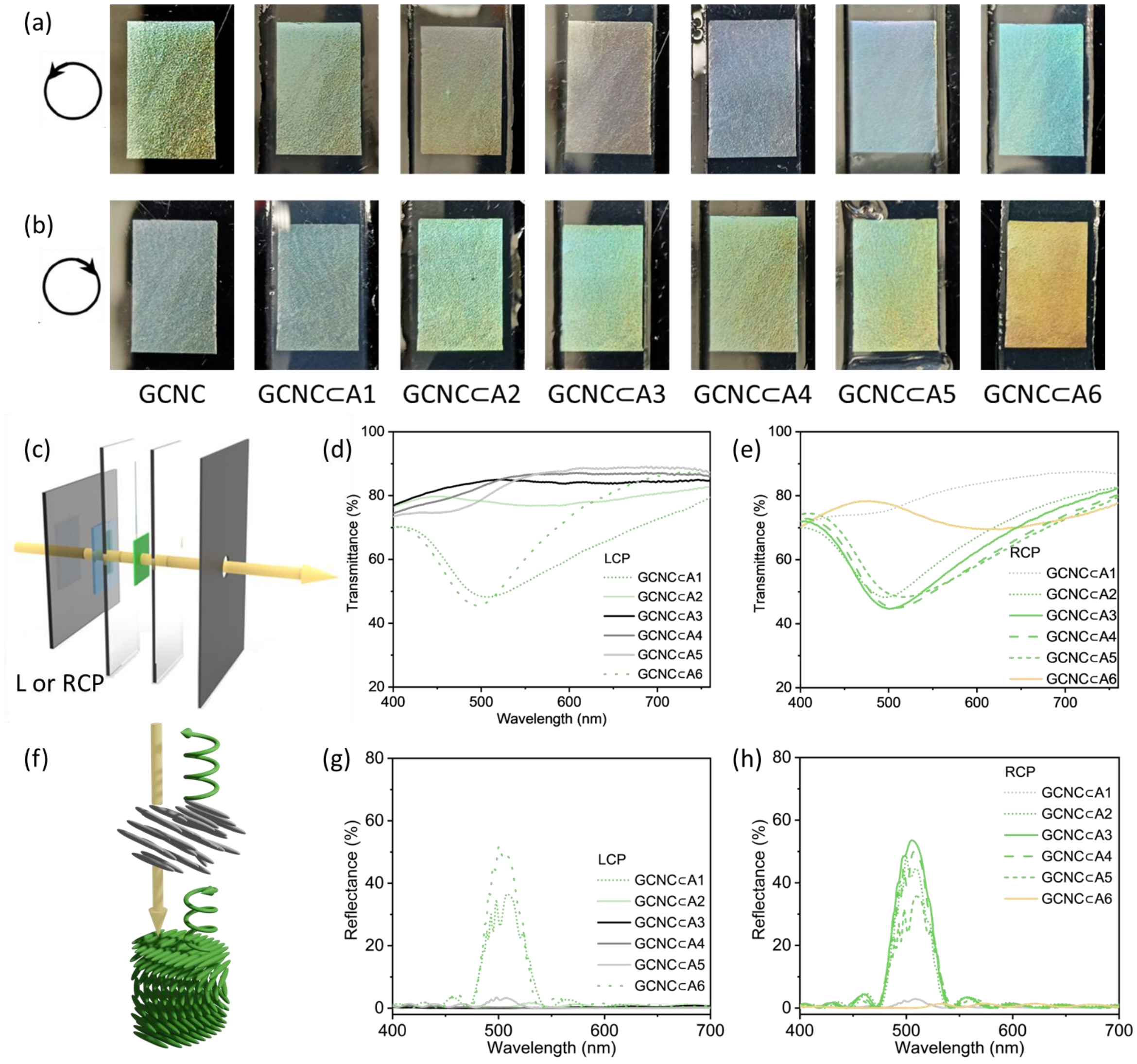

Figure 2. **Observation of R chiral reflection in chiral nematic CNC films coupled with unidirectional CNC hydrogels.** (a,b) Photographs of the chiral nematic CNC films with and without the unidirectional CNC hydrogels (**A1-A6**) on top; photos are taken through a (a) LCP and (b) RCP. (c) Schematic illustration of the setup for L-CPL or R-CPL transmission measurements of **GCNC⊂A1**, **GCNC⊂A2**, **GCNC⊂A3**, **GCNC⊂A4**, **GCNC⊂A5**, and **GCNCA⊂6**; normal light is circularly polarized by LCP or RCP. (d) Schematic illustration of the handedness inversion of the green reflected light after passing through the unidirectional hydrogel. (e,f) L-CPL (e) and R-CPL (f) transmission spectra of **GCNC⊂**A samples. (g,h) Simulated L-CPL (g) and R-CPL (h) reflectance spectra using Berreman's method. The width of the GCNC film is ~ 1.1 cm.

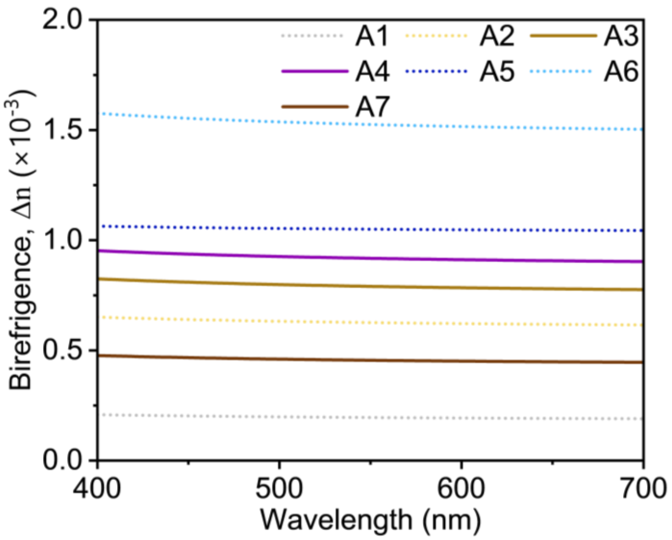

Figure S7. The birefringence dispersions for **A1-A7**.

4. It would be worthwhile to give the scalability and reproducibility more space in the manuscript, e.g.,: How uniform is retardance over centimetre areas? Does repeated wetting or drying alter Δn? Are pressures of a few newtons sufficient for devices thicker than the laboratory samples?

We performed additional experiments to evaluate the heterogeneity of the hydrogel and explore the effect of water loss on the optical behavior of the invertible chiral optical system. The average retardance is 0.488 ± 0.007 waves and the retardance at 500 nm is 0.527 ± 0.007 waves. These results indicate reasonable uniformity at the current laboratory scale. Future work could explore machine-assisted fabrication methods, such as extrusion-based 3D printing, which may promote particle alignment and improve uniformity over centimetre-scale areas. Regarding applications that require different mechanical properties, our current thin-film samples show tunable chiral optical responses under forces on the order of a few newtons. Scaling to thicker, stiffer, or more elastic materials appears feasible; however, we still need more exploration on the mechanical properties of unidirectionally aligned CNC hydrogels. Future work will focus on introducing physical cross-linkers (e.g., polyvinylpyrrolidone, PVP) or reinforcing additives, as well as tuning nanoparticle loading to engineer retardation layers with specific thickness and birefringence.

New results, with descriptions are provided here as well:

The hydrogel heterogeneity was roughly assessed by comparing the retardance at 500 nm measured in the top, center, and bottom regions of the sample. (**Figure S15**). (Page 22)

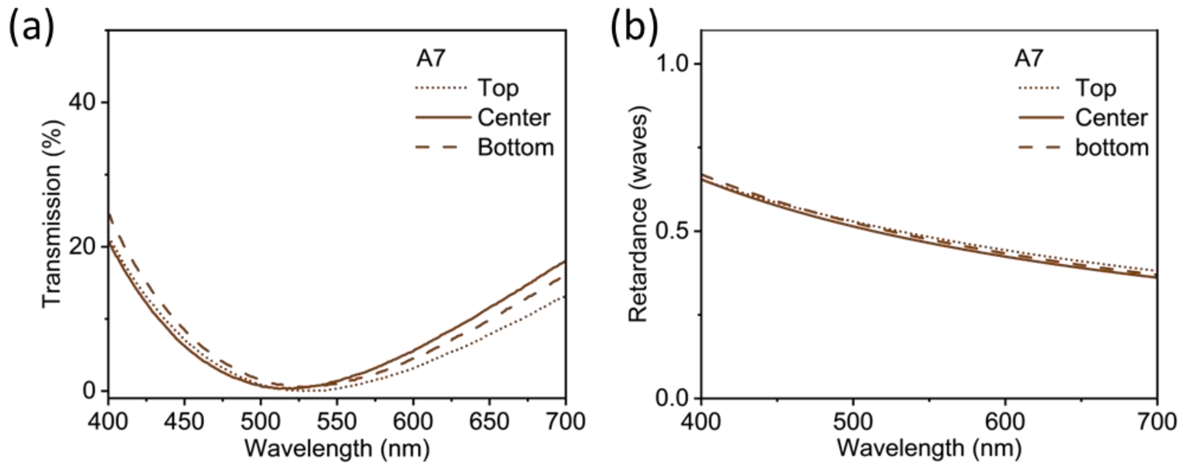

Figure S15. (a) Transmission spectra measured under parallel polarizers for the top, center, and bottom regions of sample **A7**. (b) Corresponding spectral retardance for the same regions. The average retardance is 0.488 ± 0.007 waves and the retardance at 500 nm is 0.524 ± 0.007 waves.

We also performed experiments to evaluate the effect of water loss on the chiral optical response. Water loss via evaporation or osmosis can shrink the hydrogel (**Figure S6**). After ~2.5 h evaporation at room temperature, **A3** lost its broadband polarization-rotation feature and became more wavelength-selective, resembling **A6**. Consequently, handedness inversion occurred only over a restricted wavelength range, producing reflection in both left- and right-handed channels, similar to **GCNC⊂A6**. A similar trend was observed after treatment with 1 mM NaCl solution (osmotic dehydration). Nevertheless, the loss of the broadband feature was reversible upon re-swelling the hydrogel in water. (Page 10)

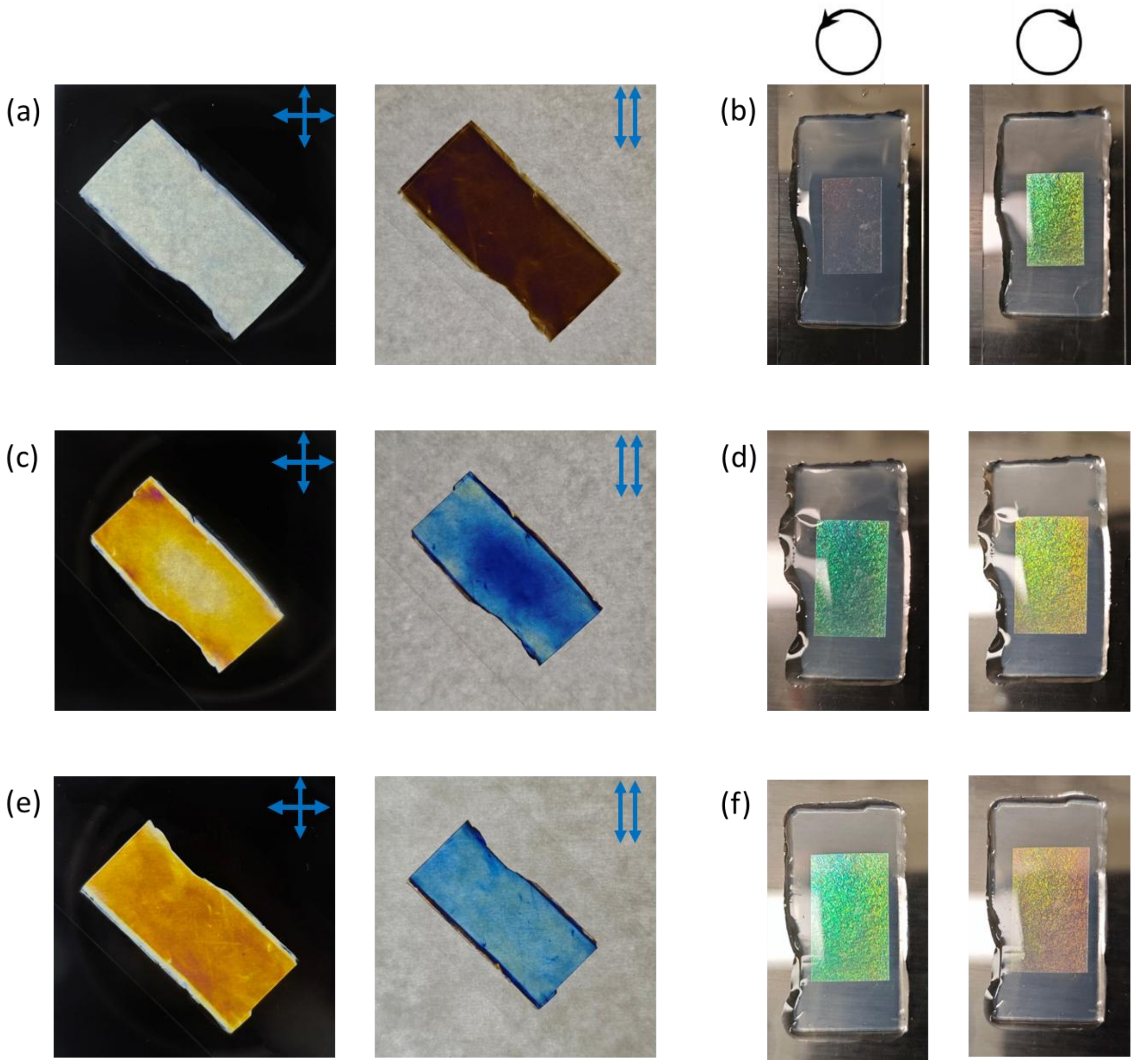

Figure S6. (a,c,e) Transmission images of hydrogel **A7** recorded under crossed polarizers (left) and parallel polarizers (right): (a) as prepared (no water evaporation), (c) after water evaporation at room temperature for 2.5 h, and (e) after treatment with 1 mM NaCl solution. (b,d,f) Photographs of the chiral nematic CNC films combined with the corresponding unidirectional CNC hydrogels shown in (a,c,e). Images were acquired through a left-handed circular polarizer (left) and right-handed circular polarizer (right).

5. Furthermore, some text in the figures is too small to read and/or cropped. Please correct.

We thank the reviewer for his/her careful review of the manuscript. We have proofread the paper again and corrected some typos.

REVIEWER 2

This work focuses on controlling the handedness of reflected light by enabling switching between left- and right-handed chiral reflections using CNC-based materials. A CNC hydrogel was developed and employed as a responsive retardation layer, enabling the conversion of left-handed chiral reflections from chiral CNC nematic films into right-handed chiral reflections. The optical properties of this bio-based unidirectional structure were found to be pressure-sensitive, which the authors attributed to thickness variations or disruptions in the alignment of the birefringent layer. The results presented are interesting, and after minor revision, the manuscript should be accepted.

We thank the reviewer for their positive assessment and for the helpful suggestions. We have addressed the questions below.

1. The formulation of A6 and A5 is very similar; the main difference is the quantity of CNCs (55 mg/mL for A5 and 57 mg/mL for A6). Can the authors explain why A6 is so selective ("…only alters the handedness of certain wavelengths of light (i.e., light in the orange and red spectral region") compared, for example, with A5?

A3 and **A4** can introduce phase shift of ~0.25 to 0.75 waves over 400-700 nm; hydrogel **A5** shows a similar retardance window, but over 425–700 nm. Because this phase-delay range spans almost the entire visible spectrum, **A3**, **A4**, and **A5** can invert the chiral reflection from the underlying CNC films over a broad wavelength range. In contrast, hydrogel **A6** introduces a phase shift of ~0.25–0.75 waves only in the orange–red region (610–700 nm). In the blue–green region (400–560 nm), the phase delay exceeds 0.75 waves; therefore, the green chiral reflection from the CNC film remains predominantly left-handed, whereas the small orange–red portion is inverted. As a result, **GCNC⊂A6** displays reflection in both left- and right-handed circular polarization channels.

We have added additional discussion to clarify the differences between the behaviors of **A5** and **A6**, as follows:

Interestingly, by carefully adjusting the particle concentration or hydrogel thickness, the unidirectional hydrogels (**A2**–**A5** and **A7**) can exhibit a uniform white transmission color (**Figure 1d**) when viewed between crossed polarizers (the hydrogel was placed at 45° between two crossed polarizers). The corresponding transmission spectra show a very broad peak spanning the visible region. These results suggest that these hydrogels can rotate the polarization of incident linearly polarized light over a broad range of wavelengths, enabling light of various wavelengths within the visible spectrum to pass through the second polarizer (i.e., the analyzer, **Figure 1g**). Further increasing the total particle concentration gives hydrogels (**A6**) that show interference color at specific wavelengths, indicating that the polarization rotation of incident linearly polarized light occurs over relatively narrower spectral bands. (Page 6)

This phenomenon can be ascribed to the retardance differences of **A6** across the visible spectrum. In the orange and red spectral region (610-700 nm), the phase shift introduced by **A6** is > 0.25 waves, which results in hydrogel **A6** altering the handedness of light. In the blue–green region (400-560 nm), the phase shift is > 0.75 waves, so the reflection in this region remains left-handed and the reflection peak narrows, producing the distinct cyan color. The combined L and R circularly polarized reflection resembles that of the bare CNC film (**Figure S5**), supporting the above analysis that hydrogel **A6** only alters the handedness of certain wavelengths of light (i.e., light in the orange

and red spectral region). As an example for comparison, hydrogel **A5** introduces a phase shift of ~ 0.44 to 0.75 waves over 425-700 nm, enabling the inversion of green chiral reflection from the underlying CNC film rather than producing light reflection of both left and right handedness. (Page 9)

To also visualize how the retardation layer with varying Δn affects the chiral reflection from the ideal chiral nematic stacks (e.g., without defects or pitch variations), we performed numeric simulation (see Section 3 in supplementary information for details) by means of the 4 × 4 Berreman matrix for a multilayer model (air ┃ uniaxial materials ┃ chiral nematic stacks ┃ silica substrate).[33-35] Note that n_0 and n_e for defining the uniaxial materials is unknown; however, since Δn can be derived from Cauchy approximation (**Figure S7**), we manually set $n_0 = 1.3$ and $n_e = 1.3+\Delta n$. The simulated spectra (**Figure 2g** and **2h**) predict that **A2**, **A3**, **A4** and **A5** effectively invert the chiral reflection from the underlying chiral nematic stacks with markedly increased right-handed reflectance and strongly suppressed left-handed components, whereas **A1** and **A6** do not invert the chiral reflection, in agreement with the experimental measurements. The simulated reflection bands are noticeably narrower than the experimental spectra. This can be ascribed to the CNC film's heterogeneities, including defect formation and pitch-length variations across the film thickness (e.g., due to drying dynamics, gravity-driven gradients or flow effects), which broaden the chiral nematic stop band and yield a wider peak in measurements (this broadening also contribute to the observed orange reflection under RCP for **GCNC⊂A6**). (Page 10-11)

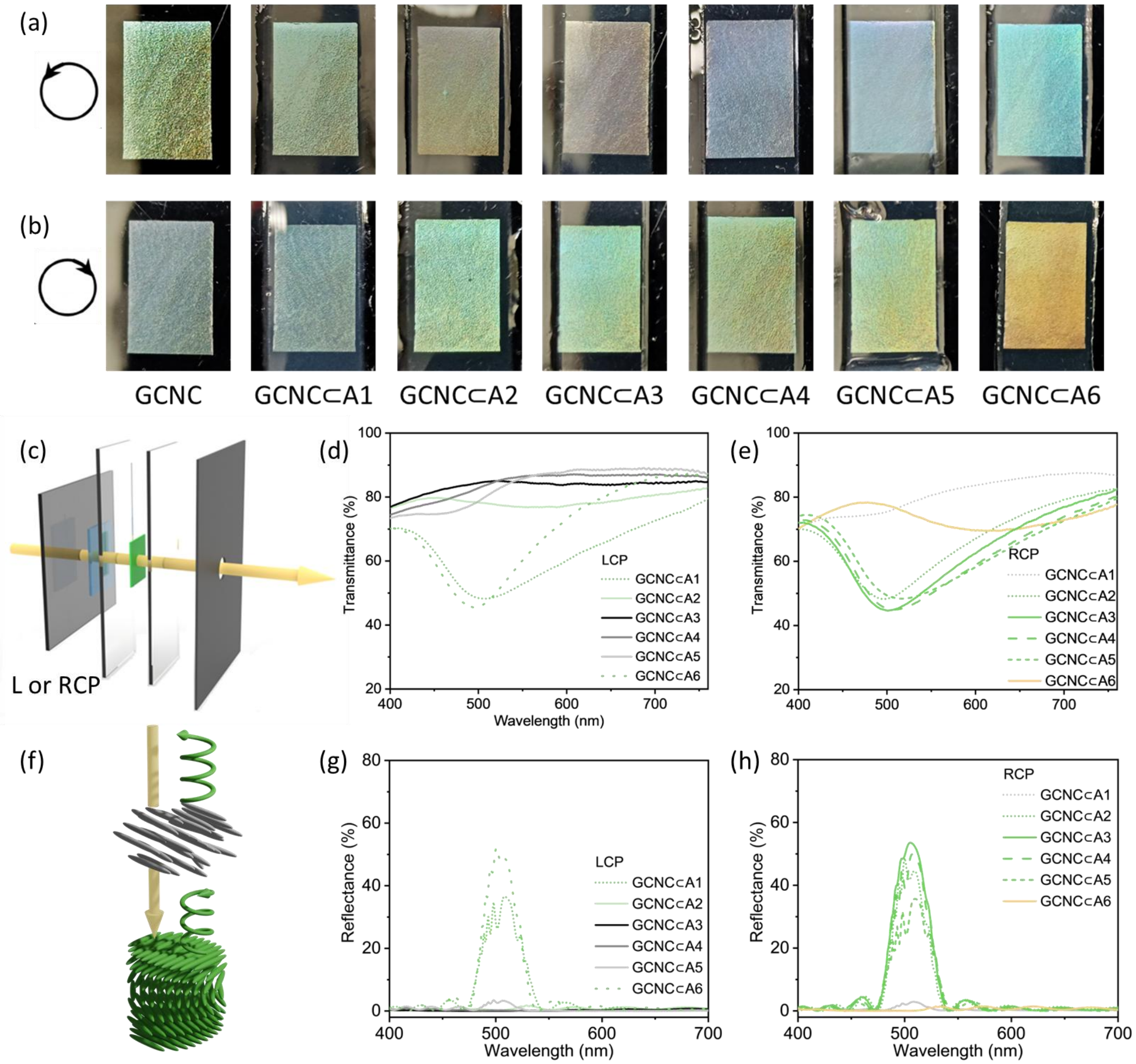

Figure 3. **Observation of R chiral reflection in chiral nematic CNC films coupled with unidirectional CNC hydrogels.** (a,b) Photographs of the chiral nematic CNC films with and without the unidirectional CNC hydrogels (**A1-A6**) on top; photos are taken through a (a) LCP and (b) RCP. (c) Schematic illustration of the setup for L-CPL or R-CPL transmission measurements of **GCNC⊂A1**, **GCNC⊂A2**, **GCNC⊂A3**, **GCNC⊂A4**, **GCNC⊂A5**, and **GCNCA⊂6**; normal light is circularly polarized by LCP or RCP. (d) Schematic illustration of the handedness inversion of the green reflected light after passing through the unidirectional hydrogel. (e,f) L-CPL (e) and R-CPL (f) transmission spectra of **GCNC⊂**A samples. (g,h) Simulated L-CPL (g) and R-CPL (h)

reflectance spectra using Berreman's method. The width of the GCNC film is ~ 1.1 cm.

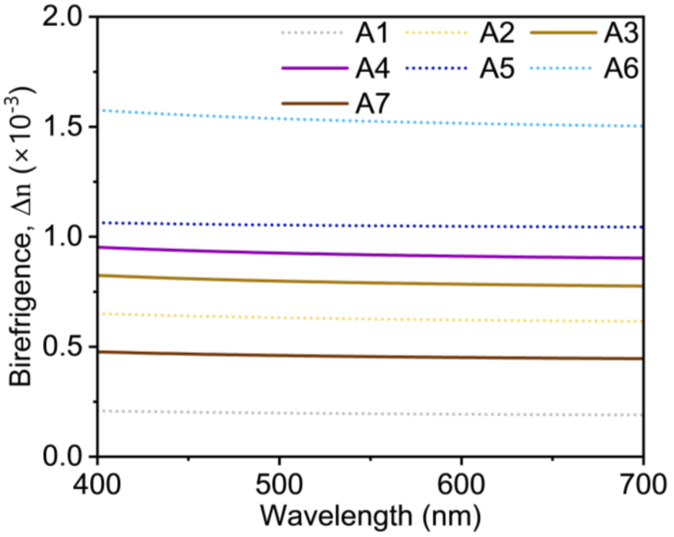

Figure S7. The birefringence dispersions for **A1-A7**.

2. Why were clay nanosheets used to increase the viscosity and not a polymer, or is the role of the clay other than increasing the viscosity? If the clays were not used, would the hydrogels present the same behaviour?

Clay for this work is hectorite-based clay nanosheets. By adjusting their concentration, these nanosheets can form transparent viscous liquids or gels and therefore do not compromise the optical transparency of the hydrogel. The addition of clay nanosheets generates higher complex viscosity and higher storage modulus across the measured frequency range compared with the clay-free system. This indicates that the clay not only increases viscosity, but also forms a reinforced physical network that enhances elasticity and suppresses post-shear relaxation of the aligned CNC domains (see Figure S2 in response to Comment 3).

We agree that polymers could also be used to tune viscosity and thereby facilitate shear-assisted alignment. In this work, we selected clay because it provides strong rheological enhancement (both viscosity and elasticity) at relatively low additive loadings while maintaining optical transparency. This allows us to keep the CNC content within a range suitable for producing unidirectional materials with broadband optical performance. It would be a nice future study to systematically compare clay- and polymer-modified formulations to quantify differences in rheology, alignment retention, and optical properties.

Regarding formulations without clay, during our studies, effective shear alignment required relatively high CNC concentrations (> 9.5 wt%). However, the resulting unidirectional hydrogels exhibited pronounced interference colors at specific wavelengths (e.g., similar to **A6**) rather than broadband optical features, particularly for thicker samples. Therefore, under our processing conditions we do not expect clay-free hydrogels at comparable solids content to exhibit the same alignment stability and broadband optical behavior. We anticipate that pure-CNC systems may be

optimized for broadband retardation layers by tuning CNC dimensions and surface charge, and we will explore this direction in future work.

3. For the preparation of the unidirectional CNC hydrogel "A microscope slide was pressed down on top of the hydrogel precursor to shear the mixture, sliding it back and forth along the long axis for about 3 mins." What was the value of the shear rate applied? Rheological measurements performed during both shear and cross-linking would be valuable to elucidate the role of the clay nanosheets further.

The shear rate is about 1.25 cm/s. We performed rheology experiments to show the role of clay nanosheets in the precursor systems. Results with additional description are provided here as well:

A microscope slide was pressed down on top of the hydrogel precursor to shear the mixture, sliding it back and forth along the long axis for about 3 mins at a rate of ~ 1.25 cm/s. (Page 22)

Rheology experiments (**Figure S2**) show that introducing clay nanosheets increases the complex viscosity and loss/storage modulus of the precursor, suggesting a more flow-resistant, elastically reinforced matrix that better restricts particle rotational/translational mobility and thus helps preserve shear-induced orientation during subsequent polymerization. During polymerization, the clay-filled system maintains higher moduli, reflecting progressive network formation in which clay promotes earlier, and stronger development of an elastic network compared with the clay-free formulation. (Page 5)

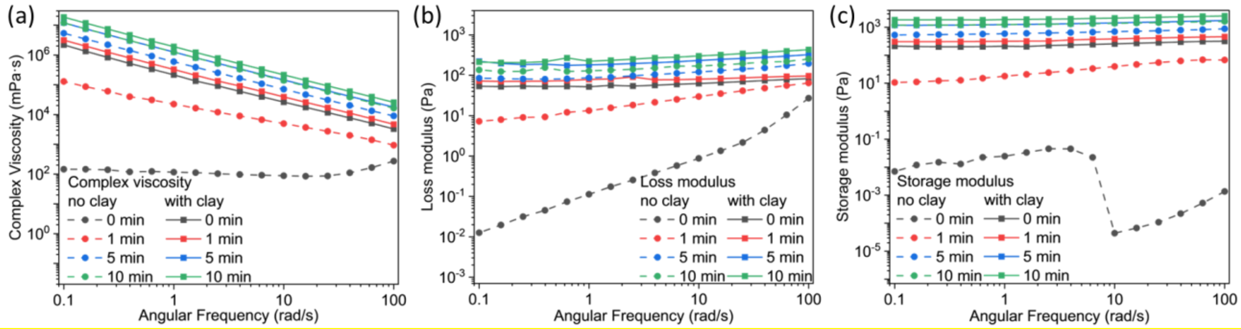

Figure S2. Frequency-dependent (a) complex viscosity, (b) loss modulus and (c) storage modulus for **A3** (■ with straight line) and **A3** without clay nanosheets (● with dash line) before and during polymerization.

REVIEWER 3

The present study reports the development of CNC-based materials exhibiting right-handed reflections, achieved using CNC hydrogels with aligned CNCs that have been cross-linked. The alignment and the structural parameters of the hydrogels strongly influence the colors. The study is clearly written and easy to follow. Before publication I have the following suggestions of improvement.

We thank the reviewer for the clear and constructive recommendations. We addressed each point below.

Major:
1. I recommend that the authors move Table S1 (or parts of Table S1) into the main text for easier comparison of the samples. In addition, how do the differences between samples relate to their optical properties?

We have added text that describes the differences in optical properties among these samples, as follows:

Interestingly, by carefully adjusting the particle concentration or hydrogel thickness, the unidirectional hydrogels (**A2**–**A5** and **A7**) can exhibit a uniform white transmission color (**Figure 1d**) when viewed between crossed polarizers (the hydrogel was placed at 45° between two crossed polarizers). The corresponding transmission spectra show a very broad peak spanning the visible region. These results suggest that these hydrogels can rotate the polarization of incident linearly polarized light over a broad range of wavelengths, enabling light of various wavelengths within the visible spectrum to pass through the second polarizer (i.e., the analyzer, **Figure 1g**). In contrast, further increasing the total particle concentration gives hydrogels (**A6**) that show interference color at specific wavelengths, indicating that the polarization rotation of incident linearly polarized light occurs over relatively narrower spectral bands. (Page 6)

This phenomenon can be ascribed to the retardance differences of **A6** across the visible spectrum. In the orange and red spectral region (610-700 nm), the phase shift introduced by **A6** is > 0.25 waves, which results in hydrogel **A6** altering the handedness of light. In the blue–green region (400-560 nm), the phase shift is > 0.75 waves, so the reflection in this region remains left-handed and the reflection peak narrows, producing the distinct cyan color. The combined L and R circularly polarized reflection resembles that of the bare CNC film (**Figure S5**), supporting the above analysis that hydrogel **A6** only alters the handedness of certain wavelengths of light (i.e., light in the orange and red spectral region). As an example for comparison, hydrogel **A5** introduces a phase shift of ~ 0.44 to 0.75 waves over 425-700 nm, enabling the inversion of green chiral reflection from the underlying CNC film rather than producing light reflection of both left and right handedness. (Page 9)

2. Regarding alignment through shearing, does this work uniformly regardless of sample thickness, or are there heterogeneities across the thickness?

We performed additional experiments, including spectroscopic measurements and relatively low magnification SEM images to evaluate the heterogeneity of the hydrogel. The average retardance is 0.488 ± 0.007 waves and the retardance at 500 nm is 0.527 ± 0.007 waves. These results indicate reasonable uniformity at the current laboratory scale. Future work could explore machine-assisted

fabrication methods, such as extrusion-based 3D printing, which may promote particle alignment and improve uniformity.

New results, with descriptions are provided here as well:

The hydrogel heterogeneity was roughly assessed by comparing the retardance at 500 nm measured in the top, center, and bottom regions of the sample (**Figure S15**). (Page 22)

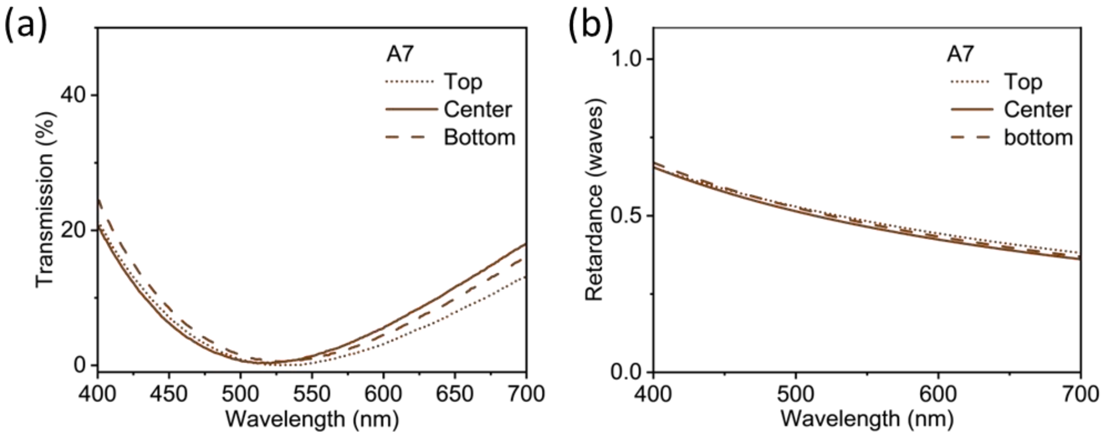

Figure S15. (a) Transmission spectra measured under parallel polarizers for the top, center, and bottom regions of sample **A7**. (b) Corresponding spectral retardance for the same regions. The average retardance is 0.488 ± 0.007 waves and the retardance at 500 nm is 0.524 ± 0.007 waves.

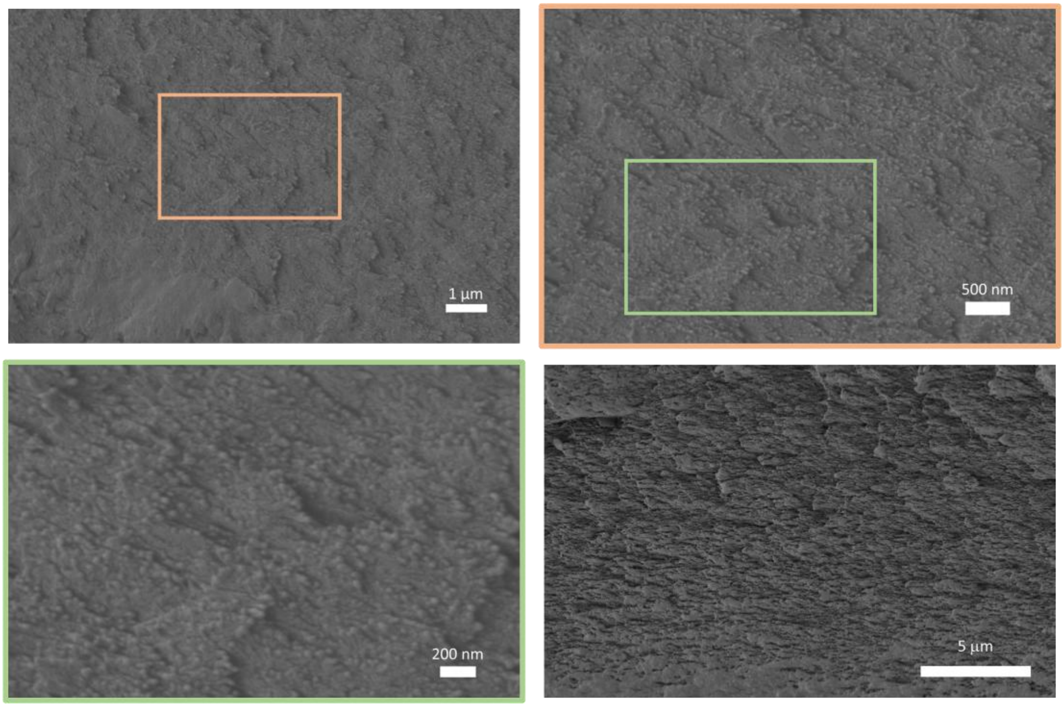

Figure S3. Cross-sectional SEM images of **A3**.

3. The spectra in figure 2e and 2f should be simulated taken into account the optical characteristics of the hydrogel.

We added simulation results and additional descriptions:

To also visualize how the retardation layer with varying Δn affects the chiral reflection from the ideal chiral nematic stacks (e.g., without defects or pitch variations), we performed numeric simulation (see Section 3 in supplementary information for details) by means of the 4 × 4 Berreman matrix for a multilayer model (air | uniaxial materials | chiral nematic stacks | silica substrate).[33-35] Note that n_0 and n_e for defining the uniaxial materials is unknown; however, since Δn can be derived from Cauchy approximation (**Figure S7**), we manually set $n_0 = 1.3$ and $n_e = 1.3+\Delta n$. The simulated spectra predict (**Figure 2g** and **2h**) that **A2**, **A3**, **A4** and **A5** effectively invert the chiral reflection from the underlying chiral nematic stacks with markedly increased right-handed reflectance and strongly suppressed left-handed components, whereas **A1** and **A6** do not invert the chiral reflection, in agreement with the experimental measurements. The simulated reflection bands are noticeably narrower than the experimental spectra. This can be ascribed to the CNC film's heterogeneities, including defect formation and pitch-length variations across the film thickness (e.g., due to drying dynamics, gravity-driven gradients or flow effects), which broaden the chiral nematic stop band and yield a wider peak in measurements (this broadening also contribute to the observed orange reflection under RCP for **GCNC⊂A6**). (Page 10-11)

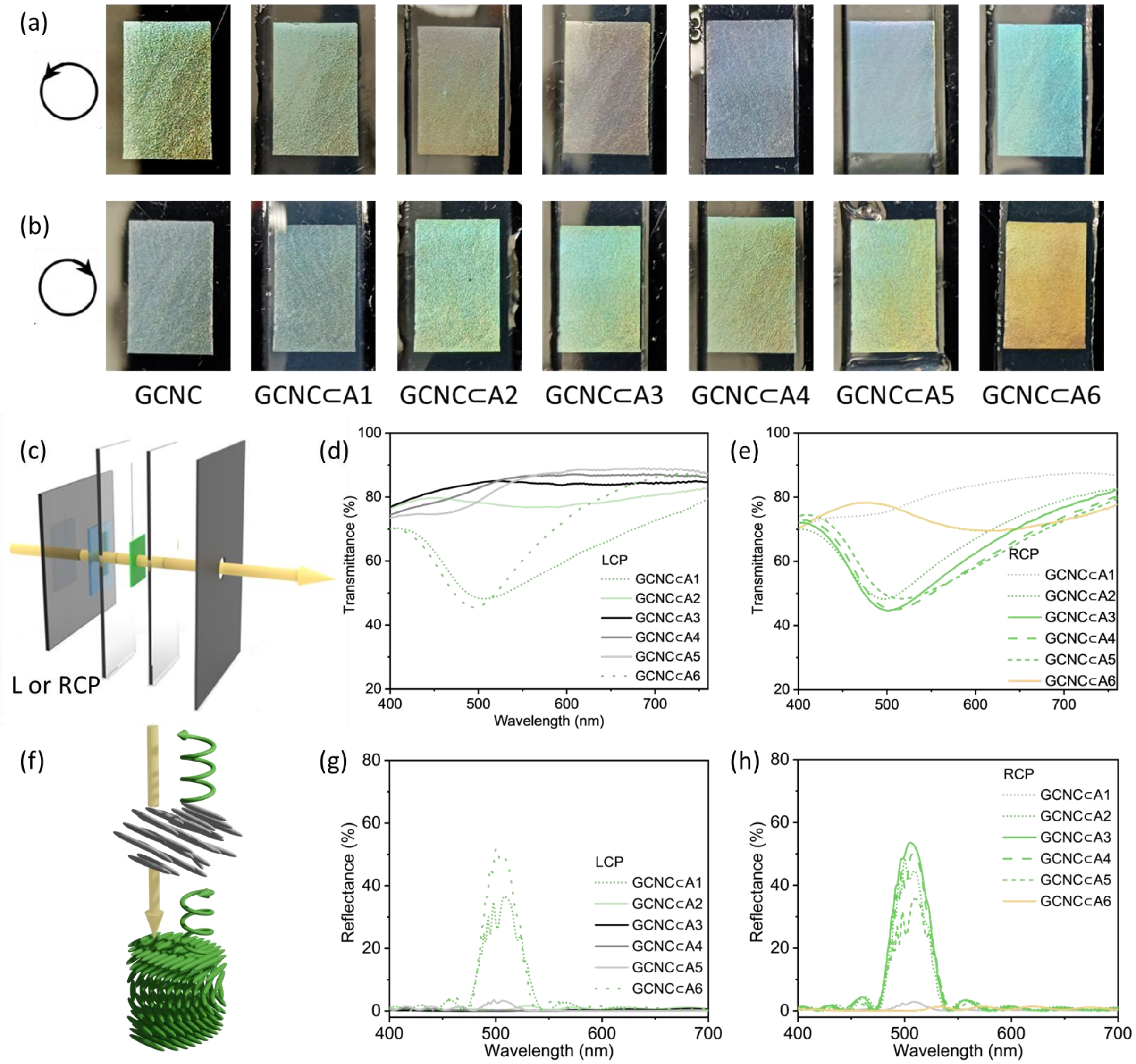

Figure 4. **Observation of R chiral reflection in chiral nematic CNC films coupled with unidirectional CNC hydrogels.** (a,b) Photographs of the chiral nematic CNC films with and without the unidirectional CNC hydrogels (**A1-A6**) on top; photos are taken through a (a) LCP and (b) RCP. (c) Schematic illustration of the setup for L-CPL or R-CPL transmission measurements of **GCNC⊂A1**, **GCNC⊂A2**, **GCNC⊂A3**, **GCNC⊂A4**, **GCNC⊂A5**, and **GCNCA⊂6**; normal light is circularly polarized by LCP or RCP. (d) A model illustrating handedness inversion of the green reflected band after passing through the unidirectional hydrogel. (e,f) L-CPL (e) and R-CPL (f) transmission spectra of **GCNC⊂**A samples. (g,h) Simulated L-CPL (g) and R-CPL (h)

reflectance spectra using Berreman's method. The width of the GCNC film is ~ 1.1 cm.

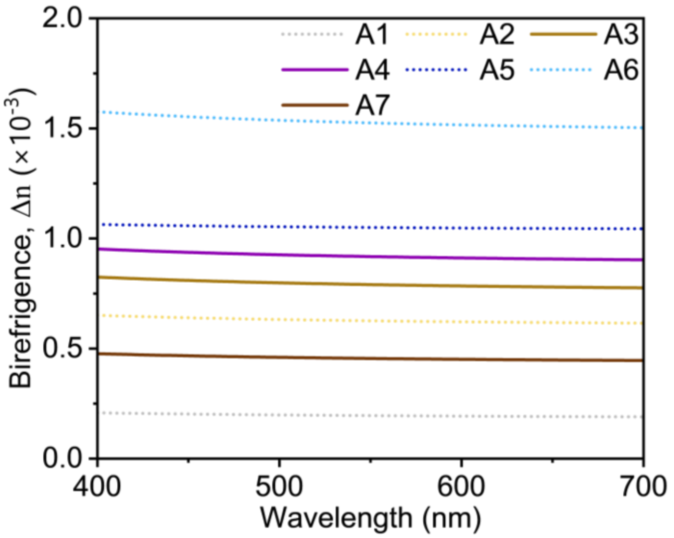

Figure S7. The birefringence dispersions for **A1-A7**.

4. Since the retardation plate is a hydrogel, it should be prone to water loss. Does this happen? How would this affect its optical properties?

We agree that investigating the effects of water loss is worthwhile, and we performed additional experiments to look at this. Here are the results and description:

We also did experiments to evaluate the effect of water loss on the chiral optical response. Water loss via evaporation or osmosis can shrink the hydrogel (**Figure S6**). After ~2.5 h evaporation at room temperature, **A3** lost its broadband polarization-rotation feature and became more wavelength-selective, resembling **A6**. Consequently, handedness inversion occurred only over a restricted wavelength range, producing reflection in both left- and right-handed channels, similar to **GCNC⊂A6**. A similar trend was observed after treatment with 1 mM NaCl solution (osmotic dehydration). Nevertheless, the loss of the broadband feature was reversible upon re-swelling the hydrogel in water. (Page 10)

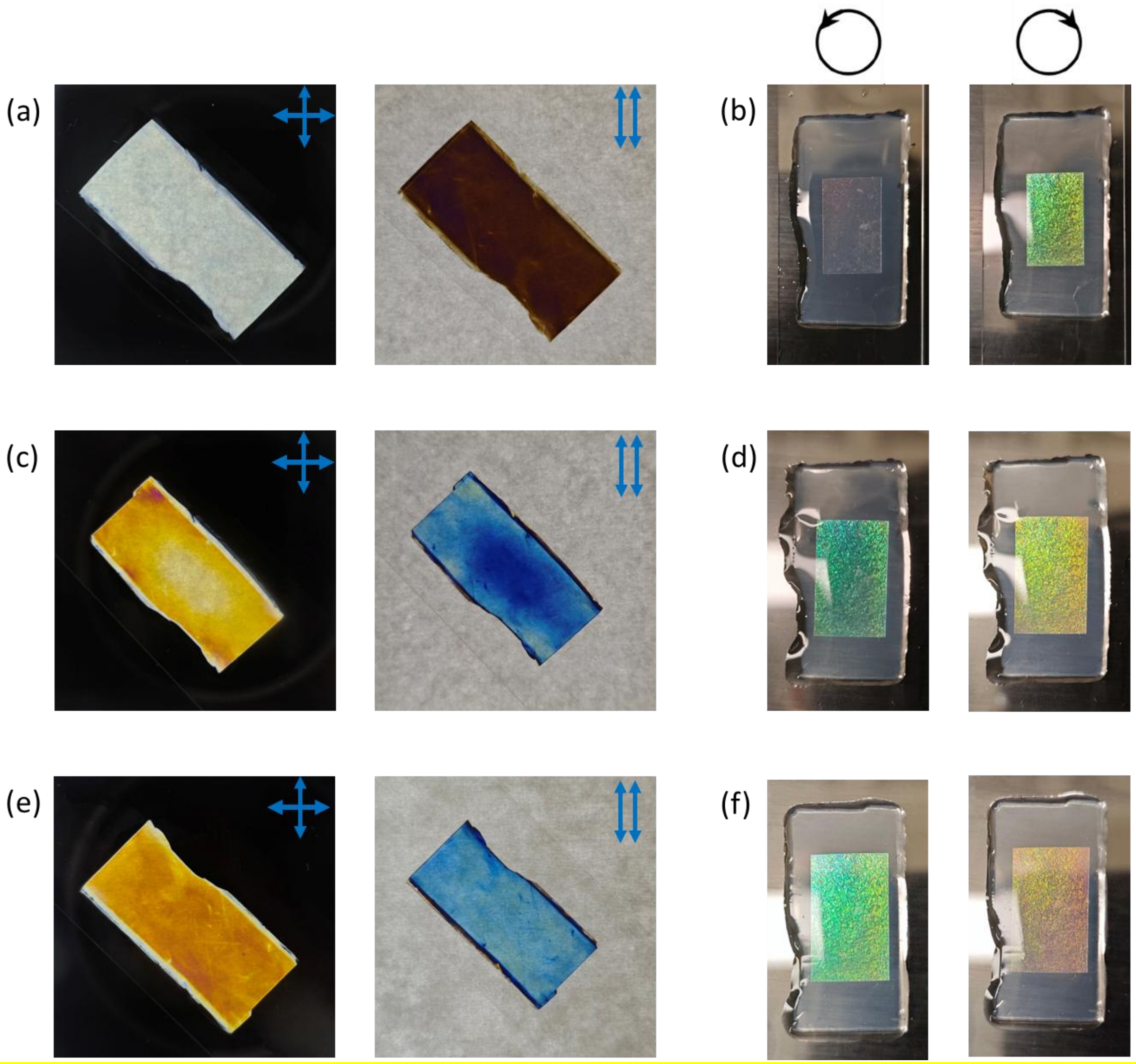

Figure S6. (a,c,e) Transmission images of hydrogel **A7** recorded under crossed polarizers (left) and parallel polarizers (right): (a) as prepared (no water evaporation), (c) after water evaporation at room temperature for 2.5 h, and (e) after treatment with 1 mM NaCl solution. (b,d,f) Photographs of the chiral nematic CNC films combined with the corresponding unidirectional CNC hydrogels shown in (a,c,e). Images were acquired through left-handed circular polarizer (left) and right-handed circular polarizer (right).

5. In Figure 6, how quickly do the materials relax back to regain their colors?

As shown in Supplementary Videos S1 and S2, the color recovers rapidly on a timescale of seconds.

Minor:

1. The font sizes in the figures should be made uniform, please compare all figures. For example, in Figure 1(f), the x- and y-axis labels are very small and hard to read. In Figure 2(e), the y-axis text is unclear, as the letter "T" is not properly reproduced.

We thank the reviewer for his/her careful review of the manuscript. We have proofread the paper again and corrected a few typos, including those above.

2. In the Experimental section, "Preparation of the unidirectional CNC hydrogel", how many times is the sliding performed, and what is the spacer distance? Please add more details to this section.

We added additional descriptions into the manuscript to provide more details, as follows:

The hydrogel precursor was then transferred to a homemade set-up (**Figure S1**) for shear alignment. The spacers were made by stacking adhesive tape to a thickness of ~300 µm for **A1–A6** and ~550 µm for **A7**. A microscope slide was pressed down on top of the hydrogel precursor to shear the mixture, sliding it back and forth along the long axis for about 3 mins at a rate of ~ 1.25 cm/s. (Page 22)

Response to Referee Reports

REVIEWER 1

The authors have substantially revised the manuscript in response to my earlier comments - thank you! The optical characterization has been expanded and is internally consistent, with broadband handedness inversion demonstrated across blue, green, and red CNC films. These additions significantly strengthen the mechanistic clarity and credibility of the work, and the presentation is much improved relative to the original submission.

That said, the study remains largely phenomenological. While the retardance window required for handedness inversion is now clearly articulated, the manuscript stops short of delivering a rigorous structure–property framework. Direct structural evidence under relevant compressive conditions is limited, and comparisons to prior waveplate-based or interlayer approaches remain qualitative - and I would think that some of this is needed for a publication in this journal.

Previous studies have reported several important strategies for generating right-handed (R) reflection in CNC-based systems, including the use of molecular liquid crystals, birefringent tape, controlled evaporation of CNC suspensions, and cellulose interlayers with multidomain nematic-like structures. However, these approaches generally did not suppress the intrinsic left-handed (L) reflection of chiral nematic CNC systems, and both L- and R-handed reflections were observed (refs. 20–24, 27, 28). Two recent interesting studies use layer-by-layer printing or assembly to produce cellulose films with right-handed helicoidal structures; however, the reflected colors were not located in the visible region (refs. 25, 26). We have mentioned these important contributions in the paper.

In the present work, we developed an elastic CNC-based retardation hydrogel layer with suitable retardance across the visible spectrum, enabling right-handed chiral reflection while strongly suppressing left-handed reflection. Furthermore, the elastic nature of the retardation layer allows pressure-dependent tuning of the optical response. This distinguishes our system from previous reports, particularly in demonstrating a CNC-based retardation material capable of chiral reflection inversion in the visible RGB range.

Following the reviewer’s suggestion, we first attempted to use electron microscopy to directly observe the CNC arrangement at the material surface. However, because the CNCs are embedded within and covered by polymer, the CNC alignment could not be clearly resolved by this method (the following figure is an example of what we typically saw).

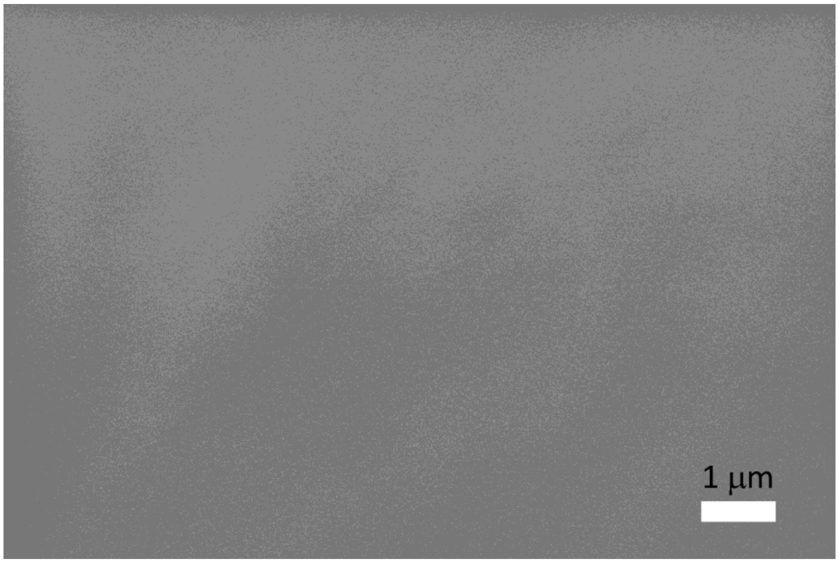

Figure: Scanning electron micrograph (SEM) of the surface of dried **A7**.

We thus turned to 2D-XRD measurements to investigate structural changes under compression. The results show that the 2D-XRD pattern remains anisotropic after compression, and the Hermans orientation factor decreasing from 0.38 to 0.31. This indicates that the unidirectional structure is maintained, but the degree of anisotropy is somewhat reduced. Because orientational birefringence generally scales with the degree of structural anisotropy/order, this reduction is expected to lower the birefringence and, consequently, the retardance. In addition, the material thickness decreases under compression, and retardance also decreases with thickness. We therefore infer that the reduction in thickness, together with the modest decrease in structural order, jointly lowers the retardance and thereby modulates the optical properties of the chiral system.

Results with descriptions are highlighted in the manuscript and provided here as well:

2D-XRD measurements were performed to assess structural changes after compression. The 2D-XRD pattern of freeze-dried **A7** prepared in the presence of a compressive force of ~4.5 N remained anisotropic, but showed a reduction in structural order (S decreases from 0.38 to 0.31, Figure S4 and S13). This suggests that the unidirectional structure is preserved after compression, but the degree of anisotropy decreases. Because orientational birefringence is generally related to the degree of structural anisotropy, this reduction is expected to lower the birefringence and, consequently, the retardance.[37] The reduced anisotropy may result from off-axis deformation (e.g., strain components not parallel to the CNC alignment) during compression. Because the material thickness also decreases under compression, and retardance scales with thickness, we assume that the reduced thickness, coupled with the decrease in structural order, may jointly lower the retardance, thereby modulating the optical properties of the chiral system.

Ref. 37. Dorohoi, D. O., *et al.* Review on Optical Methods Used to Characterize the Linear Birefringence of Polymer Materials for Various Applications. *Molecules* **28**, 2955 (2023).

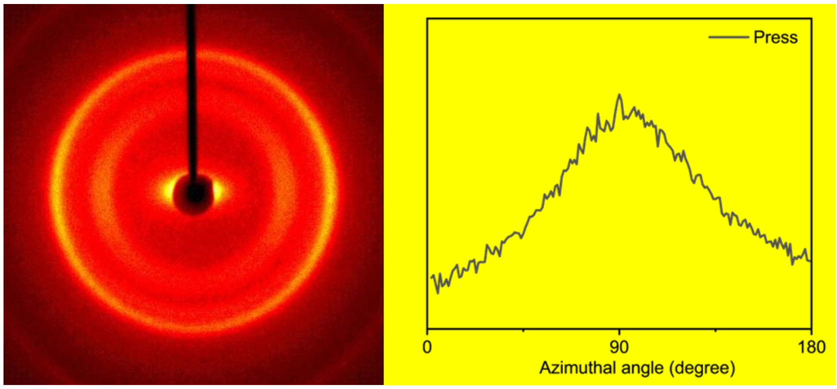

Figure S13. Two-dimensional (2D) XRD pattern (left) azimuthal-dependent intensity distributions (right) for freeze-dried **A7** under pressure.

Some other quantitative results that support the observed phenomenon are summarized here:

1). Figure 2, Figure 3, Figure 6, Figure S8 for quantification of handedness inversion and spectral position: the invertible chiral optical properties were quantified using circularly polarized light transmission spectroscopy, circularly polarized light reflection spectroscopy, circularly polarized light transmission spectroscopy, and circular dichroism spectroscopy. These measurements determine both the handedness and the wavelength range of selective reflection for the bare CNC films and for the hydrogel–film bilayers.

2). Figure 1, Figure 2, Figure S7 for quantitative link between unidirectional hydrogel optics (retardance/birefringence) and inversion outcome: We quantify the hydrogel's spectral retardance and birefringence using polarized transmission measurements coupled with a Cauchy-based dispersion treatment, yielding retardance across the visible. These data provide a predictive inversion criterion rather than a descriptive one: handedness inversion is expected when the measured phase retardation lies within the "waveplate window" of $\pi/2<\varphi<3\pi/2$ over the relevant spectral region, consistent with the observed inversion performance of A3/A4/A5/A7 and the lack (or partial) inversion for samples outside this range. The experimental trends are further consistent with Berreman-matrix simulations.

3). Figure 6, Figure S14 for quantification of the optical phenomenon under compression. we quantify retardance under applied load using the custom compression–optics setup (Fig. 6h). Under increasing compressive force, the retardance decreases and eventually falls below the inversion window; correspondingly, the system switches from the inverted (R-dominant) state back toward the intrinsic (L-dominant) state in agreement with spectral measurements. Based on the reduced degree of structural order and the reduced thickness upon compression, we think that these two factors jointly lower the retardance, thereby modulating the optical properties of the chiral system.

4). Figure 6f, Figure 2g, Figure 2h for simulation of the optical phenomenon. Berreman 4×4 matrix simulations were applied to model reflection from the multilayer stack (uniaxial retarder + chiral nematic reflector). The simulations match the experimentally observed behavior—effective inversion when φ match the experimental determined retardance values.
